# Upgrading short-read animal genome assemblies to chromosome level using comparative genomics and a universal probe set

**DOI:** 10.1101/gr.213660.116

**Published:** 2017-05

**Authors:** Joana Damas, Rebecca O'Connor, Marta Farré, Vasileios Panagiotis E. Lenis, Henry J. Martell, Anjali Mandawala, Katie Fowler, Sunitha Joseph, Martin T. Swain, Darren K. Griffin, Denis M. Larkin

**Affiliations:** 1 Department of Comparative Biomedical Sciences, Royal Veterinary College, University of London, London, NW1 0TU, United Kingdom;; 2 School of Biosciences, University of Kent, Canterbury, CT2 7NY, United Kingdom;; 3 Institute of Biological, Environmental and Rural Sciences, Aberystwyth University, Aberystwyth, SY23 3DA, United Kingdom;; 4 School of Human and Life Sciences, Canterbury Christ Church University, Canterbury, CT1 1QU, United Kingdom

## Abstract

Most recent initiatives to sequence and assemble new species’ genomes de novo fail to achieve the ultimate endpoint to produce contigs, each representing one whole chromosome. Even the best-assembled genomes (using contemporary technologies) consist of subchromosomal-sized scaffolds. To circumvent this problem, we developed a novel approach that combines computational algorithms to merge scaffolds into chromosomal fragments, PCR-based scaffold verification, and physical mapping to chromosomes. Multigenome-alignment-guided probe selection led to the development of a set of universal avian BAC clones that permit rapid anchoring of multiple scaffolds to chromosomes on all avian genomes. As proof of principle, we assembled genomes of the pigeon (*Columbia livia*) and peregrine falcon (*Falco peregrinus*) to chromosome levels comparable, in continuity, to avian reference genomes. Both species are of interest for breeding, cultural, food, and/or environmental reasons. Pigeon has a typical avian karyotype (2n = 80), while falcon (2n = 50) is highly rearranged compared to the avian ancestor. By using chromosome breakpoint data, we established that avian interchromosomal breakpoints appear in the regions of low density of conserved noncoding elements (CNEs) and that the chromosomal fission sites are further limited to long CNE “deserts.” This corresponds with fission being the rarest type of rearrangement in avian genome evolution. High-throughput multiple hybridization and rapid capture strategies using the current BAC set provide the basis for assembling numerous avian (and possibly other reptilian) species, while the overall strategy for scaffold assembly and mapping provides the basis for an approach that (provided metaphases can be generated) could be applied to any animal genome.

The ability to sequence complex animal genomes quickly and inexpensively has initiated numerous genome projects beyond those of agricultural/medical importance (e.g., Hu et al. 2009; Groenen et al. 2012) and inspired ambitious undertakings to sequence thousands of species (Zhang et al. 2014a; Koepfli et al. 2015). De novo genome assembly efforts ultimately aim to create a series of contigs, each representing a single chromosome, from p- to q- terminus (“chromosome-level” assembly). Assembling genomes using next-generation sequencing (NGS) technologies, however, typically relies on integration of the NGS data with a pre-existing chromosome-level reference assembly built with previous sequencing/mapping technologies (Larkin et al. 2012). Indeed, use of short-read NGS data rarely produces assemblies at a similar level of integrity as those provided by traditional methodologies because of (1) an inability of NGS to generate long error-free contigs or scaffolds to cover chromosomes completely and (2) a paucity of inexpensive mapping technologies to upgrade NGS genomes to chromosome level. Even for projects with sufficient read-depths and long insert libraries, software algorithms at best produce subchromosomal-sized “scaffolds” requiring physical mapping to assemble chromosomes. Newer technologies such as optical mapping (Teague et al. 2010) including BioNano (Mak et al. 2016), Dovetail (Putnam et al. 2016), and Pacific Biosciences (PacBio) long-read sequencing (Rhoads and Au 2015) provide a long-term solution to this problem. To date, however, such approaches suffer from multiple limitations; e.g., BioNano contigs do not extend across multiple DNA nick site regions, centromeres, or large heterochromatin blocks, while PacBio sequencing requires hundreds of micrograms of high-molecular-weight DNA, which is often not easy to obtain.

Bioinformatic approaches, e.g., the Reference-Assisted Chromosome Assembly (RACA) algorithm (Kim et al. 2013), were developed to approximate near chromosome-sized fragments for a de novo assembled NGS genome. RACA use requires a genome from the same clade (e.g., Order for mammals) of the target species being assembled to chromosomes (Kim et al. 2013) and sequencing of long-insert libraries. RACA produces, at best, subchromosome-sized predicted chromosome fragments (PCFs) that require further verification and subsequent chromosome assembly. It is worth mentioning that, unlike RACA, other reference-assisted assembly algorithms, e.g., Ragout (Kolmogorov et al. 2014) or Chromosomer (Tamazian et al. 2016), do not use the target genome short- and long-range paired-read data to verify synteny breaks in/between scaffolds, meaning that the target species-specific rearrangements could be missed from the reconstructed PCFs/pseudochromosomes, making the reconstructed target chromosome structures more heavily biased to the reference genome(s) than when using RACA. RACA algorithm applied to the Tibetan antelope and blind mole rat genomes significantly improved continuities of these assemblies, but they still contain more than one large PCF for most chromosomes (Kim et al. 2013; Fang et al. 2014). Therefore, a novel, integrative approach that would allow de novo assembled genomes to retain structures of the target species karyotypes is a necessity.

A dearth of chromosome-level assemblies for nearly all newly sequenced genomes limits their use for critical aspects of evolutionary and applied genomics. Chromosome-level assemblies are essential for species that are regularly bred (e.g., for food or conservation) because a known order of DNA markers facilitates establishment of phenotype-to-genotype associations for gene-assisted selection and breeding (Andersson and Georges 2004). While such assemblies are established for popular livestock species, they are not available for those species widely used in developing countries (e.g., camels, yaks, buffalo, ostrich, quail) or species bred for conservation reasons (e.g., falcons). Chromosome-level information is essential for addressing basic biological questions pertaining to overall genome (karyotype) evolution and speciation (Lewin et al. 2009). Karyotype differences between species arise from DNA aberrations in germ cells that were fixed throughout evolution. These are associated with repetitive sequences used for nonallelic homologous recombination (NAHR) in evolutionary breakpoint regions (EBRs) where ancestral chromosomes break and/or combine in descendant species genomes (Murphy et al. 2005). An alternative theory, however, suggests that proximity of DNA regions in chromatin is the main driver of rearrangements and repetitive sequences play a minor role (Branco and Pombo 2006). Regardless of the mechanism, comparisons of multiple animal genomes show that between EBRs are evolutionary stable homologous synteny blocks (HSBs). Our studies in mammals (Larkin et al. 2009) and birds (Farré et al. 2016) suggest that at least the largest HSBs are maintained nonrandomly and are highly enriched for conserved noncoding elements (CNEs), many of which are gene regulatory sequences and miRNAs (Zhang et al. 2014b). We recently hypothesized that a higher fraction of elements under negative selection involved in gene regulation and chromosome structure in avian genomes (∼7%) (Zhang et al. 2014b) compared with mammals (∼4%) (Lindblad-Toh et al. 2011) could contribute to some avian-specific phenotypes and the evolutionary stability of most avian karyotypes (Farré et al. 2016). While a high density of CNEs in avian multispecies (ms) HSBs supports this hypothesis (Farré et al. 2016), a more definitive answer might be obtained by examining the fate of CNEs in the “interchromosomal EBRs” (flanking interchromosomal rearrangements) of an avian genome with a highly rearranged karyotype.

In this study, we focused on two avian genomes. The first, the peregrine falcon (*Falco peregrinus*), has an atypical karyotype (2n = 50) (Nishida et al. 2008). The falcon's ability to fly at speeds >300 km/h and its enhanced visual acuity make it the fastest predator on earth (Tucker et al. 1998). A prolonged period of extinction risk due to persecution around World War II and secondary poisoning from organochlorine pesticides (e.g., DDT) in the 1950s–1960s (Ferguson-Lees and Christie 2005) led to its placement on the CITES list of endangered species. The second avian genome that was focused on here, the pigeon (*Columba livia*), has a typical avian karyotype (2n = 80) similar to those of reference avian genomes: chicken, turkey, and zebra finch. Pigeon is one of the earliest examples of domestication in birds (Driscoll et al. 2009) contemporarily used as food and in sporting circles (Price 2002). Pigeon breeds can vary significantly in appearance with color, pattern, head crest, body shape, feathers, tails, vocalization, and flight display variations (Price 2002), inspiring considerable interest in identifying the genetic basis for these variations (Stringham et al. 2012; Shapiro et al. 2013). For the above reasons, both species' genomes were sequenced (Shapiro et al. 2013; Zhan et al. 2013); however, their assemblies are highly fragmented and chromosome-level assemblies are thus essential.

The objective of this study was therefore to develop a novel, inexpensive, transferrable approach to upgrade fragmented genome assemblies (i.e., pigeon and falcon) to the chromosome level and to use them to address novel biological questions related to avian genome evolution. The method combines computational algorithms for ordering scaffolds into PCFs, retaining local structures of the target genome chromosomes after verification of a limited number of scaffolds, and physical mapping of PCFs directly to chromosomes with a universal set of avian bacterial artificial chromosome (BAC) probes. Studying a highly rearranged genome (falcon) compared with the avian ancestor sheds light on why interchromosomal rearrangements are infrequent in bird evolution.

## Results

**Figure 1.**
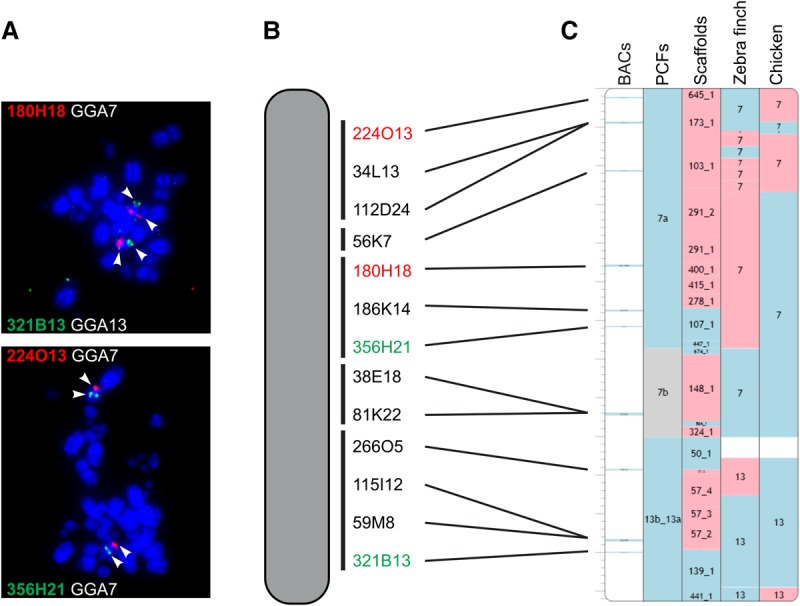
Methodology for the placement of the PCFs on chromosomes. (*A*) Dual-color FISH of universal BAC clones, (*B*) cytogenetic map of the falcon chromosome 8 (FPE8) with indication of the relative positions of the BAC clones along the chromosome, and (*C*) assembled chromosome containing PCFs 7a, 7b, and 13b_13a. Blue blocks indicate positive (+) orientation of tracks compared with the falcon chromosome; red blocks, negative (−) orientation; and gray blocks, unknown (?) orientation.

Our method involves (1) the construction of PCFs for fragmented assemblies based on the comparative and sequence read data implemented in the RACA algorithm, (2) PCR and computational verification of a limited number of scaffolds that are essential for revealing species-specific chromosome structures, (3) creation of a refined set of PCFs using the verified scaffolds and adjusted adjacency thresholds in RACA, and (4) the use of a panel of “universal” BAC clones to anchor PCFs to chromosomes in a high-throughput manner (Fig. 1).

### Construction of PCFs from fragmented assemblies

PCFs were generated for fragmented falcon and pigeon whole-genome sequences using RACA (Kim et al. 2013). For falcon, the zebra finch chromosome assembly was used as reference (divergence 60 MYA) and the chicken genome as outgroup (divergence 89 MYA). We generated a total of 113 PCFs with N50 of 27.44 Mb (Table 1). For pigeon (≥69 Myr divergence from both the chicken and zebra finch), chicken was used as reference and zebra finch as outgroup because (1) fewer pigeon scaffolds were split in this configuration (Supplemental Table S1) and (2) the high similarity of pigeon and chicken karyotypes (Derjusheva et al. 2004). This resulted in 150 pigeon PCFs with N50 of 34.54 Mb (Table 1). These initial PCF sets contained 72 (15.06%) and 78 (13.64%) scaffolds for falcon and pigeon, respectively, that were split by RACA due to insufficient read and/or comparative evidence to support their structures.

**Table 1.**
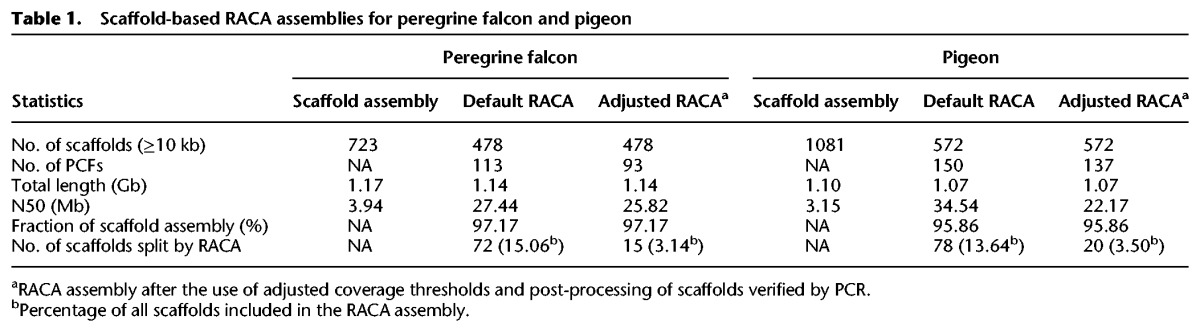
Scaffold-based RACA assemblies for peregrine falcon and pigeon

### Verification of scaffolds essential for revealing species-specific chromosome architectures

All scaffolds split by RACA contained structural differences between the target and reference chromosomes, suggesting their importance for revealing the architecture of target species chromosomes. The structures of these scaffolds were tested by PCR amplification across all the split regions defined to <6 kb in the target species scaffolds. Of these, 41 (83.67%) and 58 (84.06%) resulted in amplicons of expected length in pigeon and falcon genomic DNA, respectively (Supplemental Table S2). For the split regions with negative PCR results, we tested an alternative (RACA-suggested) order of the flanking syntenic fragments (SFs). Out of these, amplicons were obtained for 2/4 in falcon and 7/7 in pigeon, confirming the chimeric nature of the original scaffolds properly detected in these cases (Supplemental Table S2). To estimate which of the remaining split regions (>6 kb; 36 in falcon and 40 in pigeon PCFs) were likely to be chimeric, we empirically identified two genome-wide minimum physical coverage (Meyerson et al. 2010) levels, one for falcon and one for pigeon, in the SFs joining regions for which (and higher) the PCR results were most consistent with RACA predictions. If the new thresholds were used in RACA without additional scaffold verification (e.g., by PCR) or mapping data, they would lead to splitting of nearly all scaffolds with large structural misassemblies in falcon, and ∼6% of them would still be present in pigeon PCFs. The number of scaffolds containing real structural differences with the reference chromosomes that would still be split by RACA was estimated as ∼56% in the falcon and ∼43% in pigeon PCFs (Supplemental Table S2). To reduce the number of the real structural differences split in the final PCF set, PCR verification of selected scaffolds and use of independent (cytogenetic) mapping have been introduced.

### Creation of a refined set of pigeon and falcon PCFs

For new reconstructions, the adjusted physical coverage thresholds were used. In addition, we kept intact those scaffolds confirmed by PCR but split those shown to be chimeric and/or disagreeing with the cytogenetic map (see below), resulting in a total of 93 PCFs with N50 25.82 Mb for falcon and 137 PCFs with N50 of 22.17 Mb for pigeon, covering 97.17% and 95.86% of the original scaffold assemblies, respectively (Table 1). The falcon RACA assembly contained six PCFs homeologous to complete zebra finch chromosomes (TGU4A, 9, 11, 14, 17, and 19), while five pigeon PCFs were homeologous to complete chicken chromosomes (GGA11, 13, 17, 22, and 25). Only 3.50% of the original scaffolds used by RACA were split in the pigeon and 3.14% in falcon final PCFs (Table 1). The accuracy for the PCF assembly was estimated as ∼85% for falcon and ∼89% for pigeon based on the ratio of the number of SFs to the number of scaffolds (Kim et al. 2013).

### Construction of a panel of comparatively anchored BAC clones designed to hybridize in phylogenetically divergent avian species and link PCFs to chromosomes

Initial experiments on cross-species BAC mapping using fluorescence in-situ hybridization (FISH) on five avian species with divergence times between 28 and 89 Myr revealed highly varying success rates (21%–94%), with hybridizations more likely to succeed on species closely related to that of the BAC origin (Table 2). To minimize the effect of evolutionary distances between species on hybridizations, genomic features that were likely to influence hybridization success were measured in chicken, zebra finch, and turkey BAC clones (Supplemental Tables S3, S4). The classification and regression tree (CART) approach (Loh 2011) was applied to the 101 randomly selected BAC clones (Table 2). The obtained classification shows 87% agreement with FISH results (Supplemental Fig. S1). Correlating DNA features with actual cross-species FISH results led us to develop the following criteria for the selection of chicken or zebra finch BAC clones very likely to hybridize on metaphase preparations of phylogenetically distant birds (≥69 Myr of divergence; where the hybridization success rate of random BAC clones was <70%): The BAC had to have ≥93% DNA sequence alignable with other avian genomes and contain at least one conserved element (CE) ≥300 bp. Instead of a long CE, the BAC could contain only short repetitive elements (<1290 bp) and CEs of at least 3 bp long (Supplemental Fig. S1; Supplemental Table S4). The hybridization success rate with distant avian species for the set of newly selected clones obeying these criteria was high (71%–94%; Table 2). The success rates for the selected chicken BAC clones only ranged 90%–94%. From these chicken clones, 84% hybridized with chromosomes of all avian species in our set (Supplemental Fig. S2).

**Table 2.**
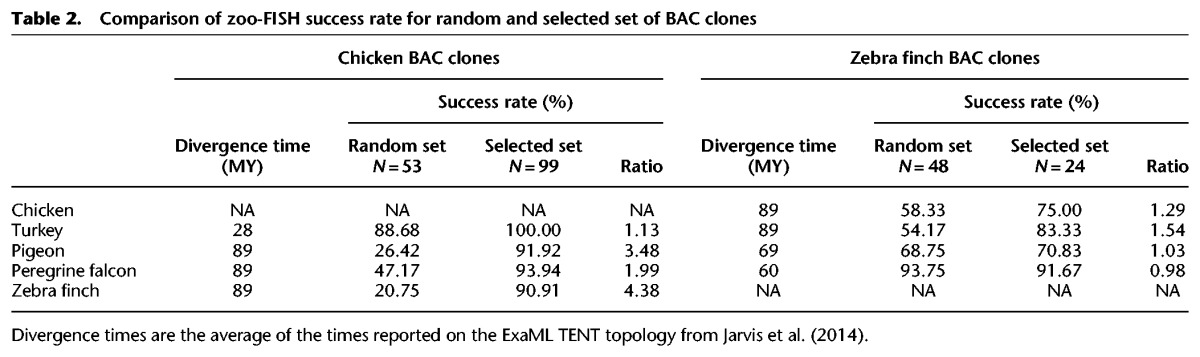
Comparison of zoo-FISH success rate for random and selected set of BAC clones

**Figure 2.**
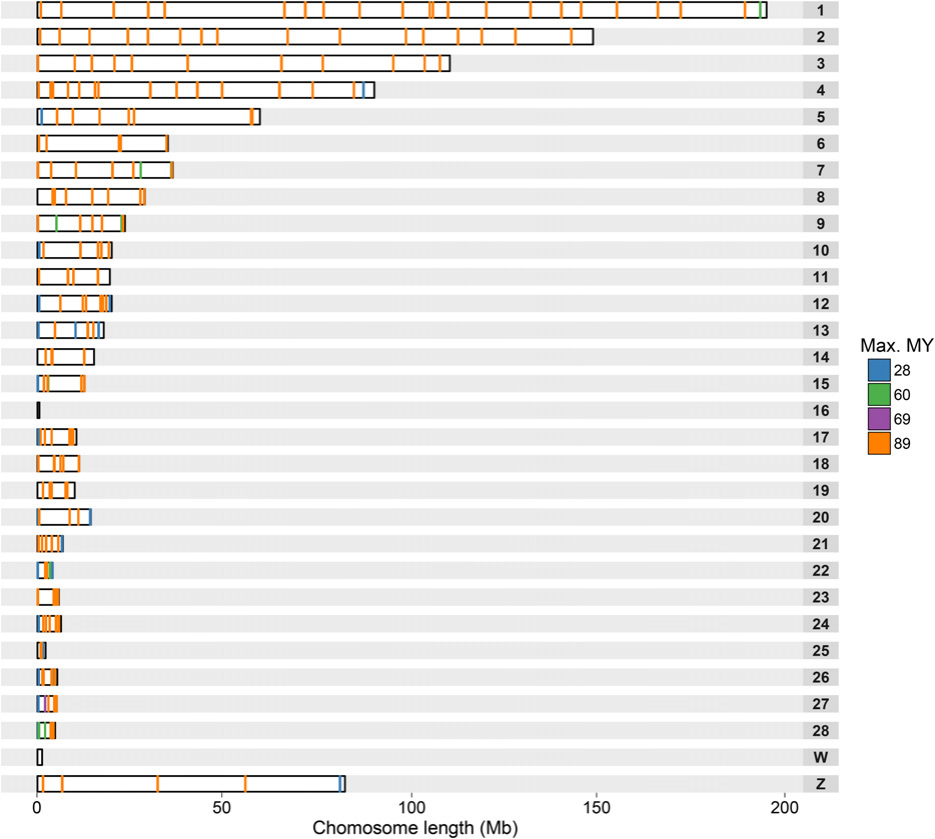
Distribution of universal BAC clones along chicken chromosomes. Each rectangle represents a chicken chromosome; the lines inside, the location of each BAC clone. BAC clones are colored accordingly to the maximum phylogenetic distance of the species they successfully hybridized. The distribution of spacing between all these BAC clones is shown on the Supplemental Figure S3.

As a final result, we generated a panel of 121 BAC clones spread across the avian genome (GGA 1-28 +Z [except 16]) that successfully hybridized across all species attempted. The collection was supplemented by a further 63 BACs that hybridized on the metaphases of at least one species that was considered phylogenetically distant (i.e., ≥69 Myr; split between Columbea and the remaining Neoavian clades) and a further 33 that hybridized on at least one other species (Fig. 2; Supplemental Table S5).

### Physical assignment of refined PCFs on the species’ chromosomes

In order to place and order PCFs along chromosomes, BAC clones from the panel described above and assigned to PCFs based on alignment results were hybridized to falcon (177 clones) and pigeon (151 clones) chromosomes (Table 3). The 57 PCFs cytogenetically anchored to the falcon chromosomes represented 1.03 Gb of its genome sequence (88% of the cumulative scaffold length). Of these, 888.67 Mb were oriented on the chromosomes (Table 3; Supplemental Table S6). The pigeon chromosome assembly consisted of 0.91 Gb in 60 pigeon PCFs representing 82% of the combined scaffold length. Of these 687.59 Mb were oriented (Table 3; Supplemental Table S7). Visualizations of both newly assembled genomes are available from the Evolution Highway comparative chromosome browser (see Supplemental Results) and our avian UCSC Genome Browser hub.

**Table 3.**
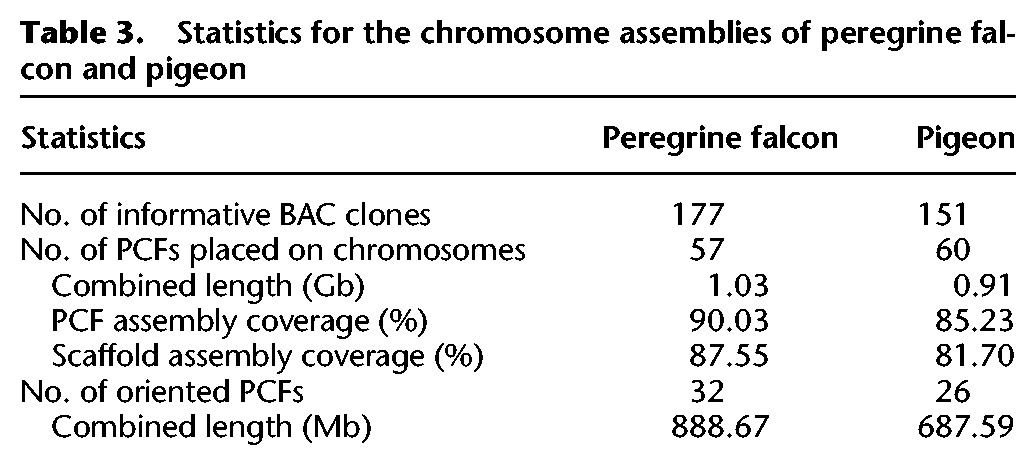
Statistics for the chromosome assemblies of peregrine falcon and pigeon

### Pigeon chromosome assembly

**Figure 3.**
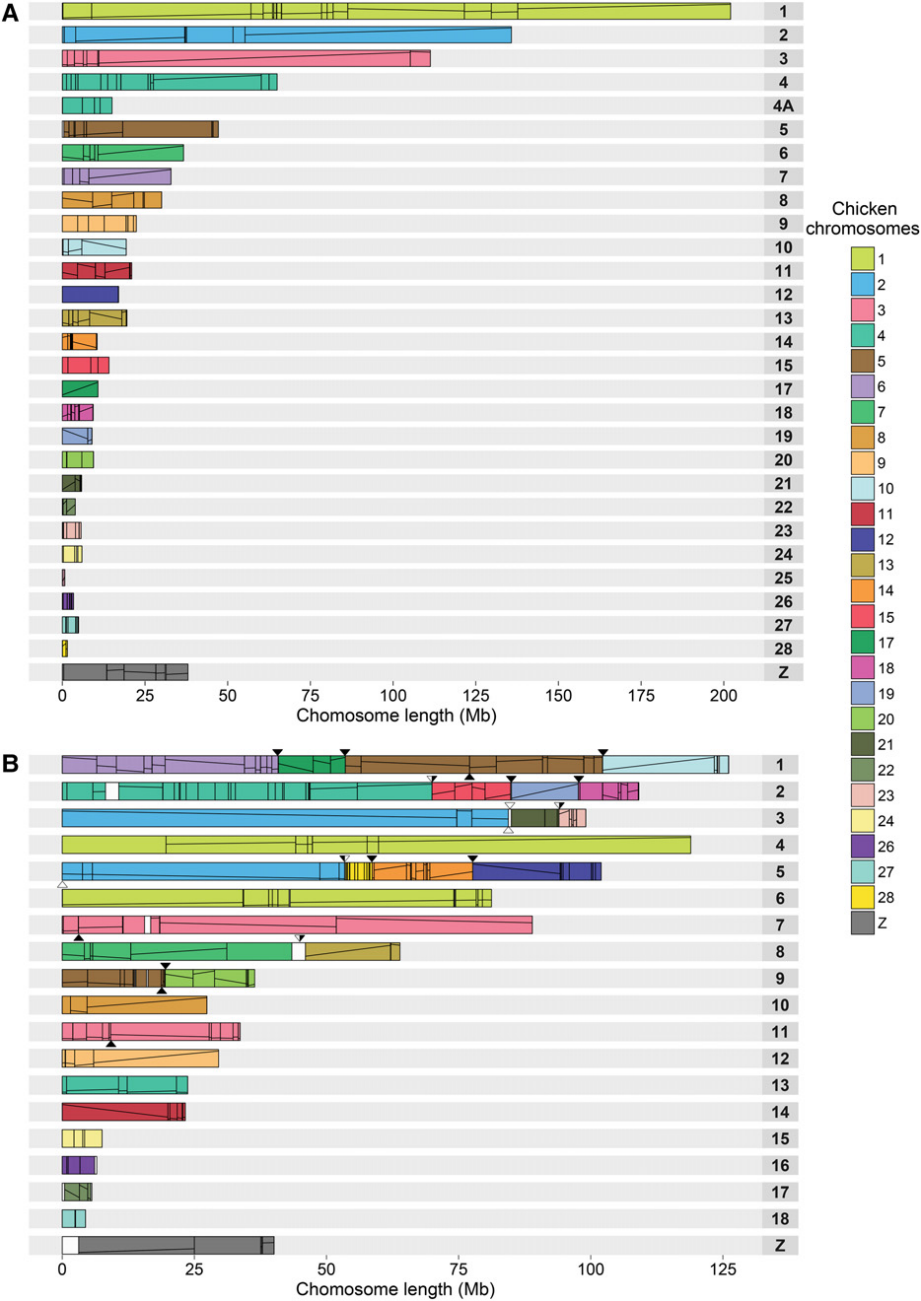
Ideogram of pigeon (*A*) and peregrine falcon (*B*) chromosomes. Numbered rectangles represent chromosomes, and colored blocks inside represent regions of homeology with chicken chromosomes. Lines within the colored blocks represent block orientation. Pigeon chromosomes 1–9 and *Z* were numbered according to the method of Hansmann et al. (2009) and the remaining chromosomes according to their chicken homeologs. Falcon chromosomes 1–13 and *Z* were numbered accordingly to the method of Nishida et al. (2008). The remaining chromosomes were numbered by decreasing combined length of the placed PCFs. Triangles above the falcon chromosomes point to the positions of falcon-specific fusions; below chromosomes, the positions of fissions. Black filling within the triangles point to the EBR boundaries used in the CNE analysis.

No deviations from the standard avian karyotype (2n = 80) were detected for pigeon with each mapped chromosome having an appropriate single chicken and zebra finch homeolog. Compared to chicken, the only interchromosomal rearrangement identified was the ancestral configuration of GGA4 found as two separate chromosomes in the pigeon and other birds (Fig. 3A; Supplemental Fig. S4; Derjusheva et al. 2004; Hansmann et al. 2009; Modi et al. 2009; http://eh-demo.ncsa.uiuc.edu/birds). Nonetheless, 70 intrachromosomal EBRs in the pigeon lineage were identified (Supplemental Table S8).

### Falcon chromosome assembly

Homeology between the chicken and the falcon was identified for all mapped chromosomes with the exception of GGA16 and GGA25 (Fig. 3B; Supplemental Fig. S5; http://eh-demo.ncsa.uiuc.edu/birds). In total, 13 falcon-specific fusions and six fissions were detected (Supplemental Table S8). Each of the chicken largest macrochromosome homeologs (GGA1 to GGA5) were split across two falcon chromosomes. Both the GGA6 and GGA7 homeologs were found as single blocks fused with other chicken chromosome material within falcon chromosomes. Among the other chicken macrochromosomes, only GGA8 and GGA9 were represented as individual chromosomes. Of the 17 mapped chicken microchromosomes, 11 were fused with other chromosomes. A total of 69 intrachromosomal EBRs were detected in the falcon lineage (Supplemental Table S8; Supplemental Results). Consistent with our previous report (Farré et al. 2016), falcon intrachromosomal EBRs were found highly enriched for the LTR-ERV1 transposable elements (TEs; *t*-test *P* < 0.05) (Supplemental Table S9). Both fusion and fission EBRs were not significantly enriched for any type of TEs.

### Fate of CNEs in avian inter- and intrachromosomal EBRs

**Figure 4.**
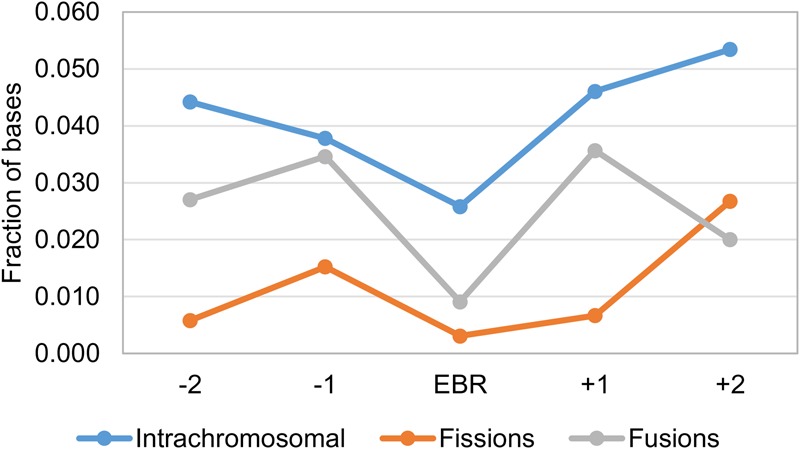
Average fraction of bases within conserved noncoding elements (CNEs) in avian EBRs and two flanking regions upstream (−) and downstream (+).

The falcon chromosome assembly provided us with a set of 19 novel interchromosomal EBRs not previously found in published avian chromosome assemblies (Fig. 3B; Supplemental Table S8). To investigate the fate of CNEs in avian EBRs, we calculated densities of avian CNEs in the chicken chromosome regions corresponding to the chicken, falcon, pigeon, flycatcher, and zebra finch intrachromosomal and interchromosomal EBRs defined to ≤100 kb in the chicken genome (Fig. 4; Supplemental Table S10). Avian EBRs had a significantly lower fraction of CNEs than their two adjacent chromosome intervals of the same size each (up- and downstream; *P* = 3.35 × 10^−7^) (Supplemental Table S11). Moreover, the interchromosomal EBRs (fusions and fissions) had, on average, approximately 12 times lower density of CNEs than the intrachromosomal EBRs (*P* = 2.40 × 10^−5^) (Supplemental Table S11). The lowest density of CNEs was observed in the fission breakpoints (*P* = 0.04) (Fig. 4; Supplemental Table S11).

To identify CNE densities and the distribution associated with avian EBRs at the genome-wide level, we counted CNE bases in 1-kb windows overlapping EBRs and avian msHSBs >1.5 Mb (Farré et al. 2016). The average density of CNEs in the EBR windows was lower (0.02) than in msHSBs (0.11). The density of CNEs in the fission EBRs was the lowest observed, without CNE bases (from now on “zero CNE windows”), while intrachromosomal EBRs were the highest among the EBR regions (0.02) (Supplemental Table S12). The genome-wide CNE density was 0.09, closer to the density observed in msHSBs. Of the ∼347 Mb of chicken genome found in the “zero CNE windows,” 0.5% was associated with EBRs and 15% with msHSBs. To investigate if these intervals are distributed differently in the breakpoint and synteny regions, we compared distances between the “zero CNE windows” and the closest window with the average msHSB CNE density or higher in EBRs, msHSBs, and genome-wide. The median of the distances between these two types of windows was the lowest in the msHSBs (∼4 kb), intermediate in the intrachromosomal (∼19 kb) and fusion EBRs (∼23 kb), and highest in the fission EBRs (∼35 kb) (Supplemental Table S13). All these values were significantly different from the genome-wide average distance of ∼6 kb (*P* < 2.2 × 10^−16^) and also significantly different from each other (*P* ≤ 0.004) (Supplemental Table S12; Supplemental Fig. S6).

## Discussion

In this study, we present a novel integrative approach to upgrade sequenced animal genomes to the chromosome level. We have previously reported a limited success with the use of high-gene density and low-repeat content BAC clones for cross-species hybridization (Larkin et al. 2006; Romanov et al. 2011). However, the use of such probes for whole-genome chromosomal assembly has not hitherto been demonstrated. That is, in this study, we made use of the whole-genome sequences from multiple species and applied a systematic approach to design a panel of universally hybridizing BAC probes along the length of each chromosome. By using these probes as a basis and in combination with comparative sequence analysis, targeted PCR, and optimized high-throughput cross-species BAC hybridizations, the approach herein presented thus represents a unique methodology to achieve chromosome-level reconstruction for scaffold-based de novo assemblies that could be applied to any animal genome provided an actively growing population of cells can be obtained to generate metaphase preparations.

In this study, we provide proof of principle for this new approach by generating such assemblies for two previously published, but highly fragmented, avian genomes. The resulting chromosome-level assemblies contain >80% of the genomes (compared with current estimates of genome size) and, in continuity, are comparable to those obtained by combining the traditional sequencing and mapping techniques (Deakin and Ezaz 2014) but require much less cost and resources. Given that it has been suggested that estimates of genome size based on cytology are inaccurate and usually overestimated (Kasai et al. 2012, 2013), techniques such as flow cytometry should be used to estimate genome size more accurately (Kasai et al. 2012, 2013). Flow cytometry will ultimately be able to determine the extent to which the genomes are actually covered by new procedures to upgrade their assemblies and will be invaluable in pointing out any remaining gaps to fill. Indeed, this approach could be augmented further by chromosome-specific DNA sequencing such as has recently been demonstrated in the B chromosomes of two deer species (Makunin et al. 2016)

Molecular and cytogenetic studies to date suggest that the majority of avian genomes remain remarkably conserved in terms of chromosome number (in 60%–70% of species 2n = ∼80) and that interchromosomal changes are relatively rare (Griffin et al. 2007; Schmid et al. 2015). Exceptions include representatives of *Psittaciformes* (parrots), *Sphenisciformes* (penguins), and *Falconiformes* (falcons). This study represents the first reconstruction of a highly rearranged avian karyotype (peregrine falcon). It demonstrates that fusion is the most common mechanism of interchromosomal change in this species, with some resulting chromosomes exhibiting as many as four fused ancestral chromosomes. There was no evidence of reciprocal translocations, and all microchromosomes remained intact, even when fused to larger chromosomes. Recently, we suggested possible mechanisms why avian genomes, with relatively rare exceptions, remain evolutionarily stable interchromosomally and why microchromosomes represent blocks of conserved synteny (Romanov et al. 2014; Farré et al. 2016). Absence of interchromosomal rearrangement (as seen in most birds) could suggest either an evolutionary advantage to retaining such a configuration or little opportunity for change. A smaller number of transposable elements in avian genomes compared with other animals would indicate that avian chromosomes indeed have fewer opportunities for chromosome merging using NAHR, explaining the presence of multiple microchromosomes. Our study provides additional support for this hypothesis as in the falcon lineage only intrachromosomal EBRs were significantly enriched in transposable elements, while interchromosomal EBRs (flanking both fusions and fissions) were not found significantly enriched. On the other hand, a strong enrichment for avian CNEs in the regions of interspecies synteny in birds and other reptiles suggests evolutionary advantage of maintaining established synteny (Farré et al. 2016), implying that fission events should be rare in avian evolution. In this study, we present the first analysis of a significant number of interchromosomal EBRs by analysis of the falcon genome, demonstrating that those rare interchromosomal rearrangements that are fixed in the avian lineage-specific evolution did indeed appear in areas of a low density of CNEs. This applies to both fission and fusion events. Our results demonstrate moreover that, to be suitable for chromosomal fission, the sites of interchromosomal EBRs are restricted further as they need to be significantly more distant from the areas with high CNE density than the equivalent intervals found in the regions of ms synteny, other EBR types, or on average in the genome. This might also explain why falcon-specific fission breakpoints appear to be reused in other avian lineages as intrachromosomal EBRs. The study of intrachromosomal changes in pigeons, falcons (this study), and Passeriform species (Skinner and Griffin 2012; Romanov et al. 2014) suggests that these events might have a less dramatic effect on *cis* gene regulation than interchromosomal events. Indeed, intrachromosomal EBRs appear in regions of significantly higher CNE density than interchromosomal EBRs. Why, then, do species such as falcons and parrots undergo wholesale interchromosomal rearrangement (previously reported) but (according to this study) with fission restricted to a few events and fusion more common? The absence of positive selection for change in chromosome number (or lack of templates for NAHR) possibly explains why there was little fixation of any interchromosomal change among birds in general (Bush et al. 1977; Fontdevila et al. 1982; Burt et al. 1999; Burt 2002); however, why this positive selection has been reintroduced (or barriers to it have been removed) in selected orders is still a matter of conjecture.

The design and use of a set of BAC probes intended to work equally well on a large number of diverged avian species created a resource for physical mapping that is transferrable to multiple species. In this regard, mammals are the greatest priority as they are the most studied phylogenetic Class of organisms in the scientific literature. Reasons for this include human interest (e.g., clinical studies), biomedical models (e.g., mouse, rat, rabbit, pig), companion animals (e.g., cat, dog), and agricultural mammals (pig, sheep, cattle, etc.). Many are on the CITES threatened/endangered list, and with impending global warming, tools for the study of ecology and conservation of these animals are a priority; many extinct species also still attract considerable interest. Of the more than 5000 extant species, however, only about 20 have genomes assembled to chromosomes (with primates, rodents, and artiodactyls disproportionally overrepresented), with more than 10 of the 26 orders having no chromosome-level assemblies at all. Recently greater than 50 de novo mammalian assemblies have been produced (more are inevitable); these, however, at best, are collections of subchromosomal-sized scaffolds. Moreover, several hundred are currently being assembled to scaffold level by individual projects or consortia such as Genome10K (Koepfli et al. 2015). Building a mammalian universal BAC set would be a greater challenge than in birds as mammalian genomes have more repetitive sequences and are about three times larger; thus, more BACs would be needed to achieve the same level of mapping resolution. On the other hand, the development of advanced mapping and sequencing techniques (e.g., Dovetail, BioNano, or PacBio) will eventually provide an opportunity to replace RACA PCFs with longer and more complete subchromosomal-sized superscaffolds or sequence contigs requiring fewer BACs to anchor them to chromosomes. The availability of large numbers of high-quality mammalian BAC clone libraries from many species makes our approach more applicable to mammals than to any other animal group. If we add the fact that our avian BAC set is showing good success rates on lizard and turtle chromosomes (data not shown), building chromosomal assemblies for all vertebrate and ultimately all animal groups supported by universal collection of BACs is a realistic objective for the near future.

## Methods

### Avian genome assemblies, repeat masking, and gene annotations

The chicken (ICGSC Gallus_gallus 4.0) (Hillier 2004), zebra finch (WUGSC 3.2.4) (Warren et al. 2010), and turkey (TGC Turkey_2.01) (Dalloul et al. 2010) chromosome assemblies were downloaded from the UCSC Genome Browser (Kent et al. 2002). The collared flycatcher (FicAlb1.5) (Ellegren et al. 2012) genome was obtained from NCBI. Scaffold-based (N50 > 2 Mb) assemblies of the pigeon, falcon, and 16 additional avian genomes were provided by the Avian Phylogenomics Consortium (Zhang et al. 2014a). All sequences were repeat-masked using Window Masker (Morgulis et al. 2006) with the *-sdust* option and Tandem Repeats Finder (Benson 1999). Chicken gene (version of 27/04/2014) and repetitive sequence (version of 11/06/2012) annotations were downloaded from the UCSC Genome Browser (Rosenbloom et al. 2015). Chicken genes with a single ortholog in the human genome were extracted from Ensembl Biomart (v.74) (Kinsella et al. 2011).

### Pairwise and multiple genome alignments, nucleotide evolutionary conservation scores, and CEs

Pairwise alignments using chicken and zebra finch chromosome assemblies as references and all other assemblies as targets were generated with *LastZ* (v.1.02.00) (Harris 2007) and converted into the UCSC “chains” and “nets” alignment formats with the Kent-library tools (Kent et al. 2003; Supplemental Methods). The evolutionary conservation scores and DNA CEs for all chicken nucleotides assigned to chromosomes were estimated using PhastCons (Siepel et al. 2005) from the multiple alignments of 21 avian genomes (Supplemental Methods). CNEs obtained from the alignments of 48 avian genomes were used (Farré et al. 2016).

### Reference-assisted chromosome assembly of pigeon and falcon genomes

Pigeon and falcon PCFs were generated using RACA (Supplemental Methods; Kim et al. 2013) tool. We chose the zebra finch genome as the reference and chicken as the outgroup for the falcon based on the phylogenetic distances between the species (Jarvis et al. 2014). For the pigeon, both chicken as reference and zebra finch as outgroup and vice versa experiments were performed as the pigeon is phylogenetically distant from the chicken and zebra finch. Two rounds of RACA were done for both species. The initial run was performed using the following parameters: *WINDOWSIZE=10 RESOLUTION=150000 MIN_INTRACOV_ PERC=5*. Prior to the second run of RACA, we tested the scaffolds split during the initial RACA run using PCR amplification across the split intervals (see below) and adjusted the parameters accordingly (Supplemental Methods).

### PCR testing of adjacent SFs

Primers flanking split SF joints within scaffolds or RACA predicted adjacencies were designed using Primer3 software (v.2.3.6) (Untergasser et al. 2012). To avoid misidentification of EBRs or chimeric joints, we selected primers only within the sequences that had high-quality alignments between the target and reference genomes and were found in adjacent SFs. Due to alignment and SF detection settings, some of the intervals between adjacent SFs could be >6 kb, and primers could not be chosen for a reliable PCR amplification. In such cases, we used CASSIS software (Baudet et al. 2010) and the underlying alignment results to narrow gaps between adjacent SFs where possible. Whole blood was collected aseptically from adult falcons and pigeons. DNA was isolated using DNeasy blood and tissue kit (Qiagen) following standard protocols. PCR amplification was performed according to the protocol described in the Supplemental Methods.

### BAC clone selection

The chromosome coordinates of chicken (CHORI-261), turkey (CHORI-260), and zebra finch (TGMCBA) BAC clones in the corresponding genomes were extracted from NCBI clone database (Schneider et al. 2013). We removed all discordantly placed BAC clones (based on BAC end sequence [BES] mappings) following the NCBI definition of concordant BAC placement. Briefly, a BAC clone placement was considered concordant when the estimated BAC length in the corresponding avian genome is within [library average length ± *3* × *standard deviation*] and BAC BESs map to the opposite DNA strands in the genome assembly. Turkey and zebra finch BAC clone coordinates were translated into chicken chromosome coordinates using the UCSC Genome Browser *liftOver* tool (Kent et al. 2002) with a minimum ratio of remapped bases >0.1.

For each BAC clone mapped to the chicken chromosomes, various genomic features selected to estimate the probability of clones to hybridize with metaphase chromosomes in distant avian species were calculated (Supplemental Table S3) using a custom Perl script or extracted from gene, repetitive sequence, CE, and nucleotide conservation score files. The clones selected for mapping experiments were originally obtained from the BACPAC Resource Center at the Children's Hospital Oakland Research Institute and the zebra finch TGMCBa library (Clemson University Genomics Institute).

### Classification tree

The classification tree was created in R (v.3.2.3) (R Core Team 2015) using the CART algorithm included in the rpart package (v.4.1-10) (https://cran.r-project.org/web/packages/rpart). We introduced an adjusted weight matrix setting: The cost of returning a false positive was twice as high as the cost of a false negative. The tree was visualized with rattle package (v.4.1.0) (Williams 2011).

### Cell culture and chromosome preparation

Chromosome preparations were established from fibroblast cell lines generated from collagenase treatment of 5- to 7-d-old embryos or from skin biopsies. Cells were cultured at 40°C and 5% CO_2_ in Alpha MEM (Fisher), supplemented with 20% fetal bovine serum (Gibco), 2% Pen-Strep (Sigma), and 1% L-glutamine (Sigma). Chromosome suspension preparation followed standard protocols; in brief, mitostatic treatment with colcemid at a final concentration of 5.0 μg/mL for 1 h at 40°C was followed by hypotonic treatment with 75 mM KCl for 15 min at 37°C and fixation with 3:1 methanol:acetic acid.

### Preparation of BAC clones for FISH

BAC clone DNA was isolated using the Qiagen miniprep kit prior to amplification and direct labeling by nick translation. Probes were labeled with Texas red-12-dUTP (Invitrogen) and FITC-fluorescein-12-UTP (Roche) prior to purification using the Qiagen nucleotide removal kit.

### FISH

Metaphase preparations were fixed to slides and dehydrated through an ethanol series (2 min each in 2× SSC, 70%, 85%, and 100% ethanol at room temperature). Probes were diluted in a formamide buffer (Cytocell) with chicken hybloc (Insight Biotech) and applied to the metaphase preparations on a 37°C hotplate before sealing with rubber cement. Probe and target DNA were simultaneously denatured on a 75°C hotplate prior to hybridization in a humidified chamber for 72 h at 37°C. Slides were washed post-hybridization for 30 sec in 2× SSC/0.05% Tween 20 at room temperature and then counterstained using VECTASHIELD anti-fade medium with DAPI (Vector Labs). Images were captured using an Olympus BX61 epifluorescence microscope with cooled CCD camera and SmartCapture (Digital Scientific UK) system. In selected experiments, we used multiple hybridization strategies, making use of the Cytocell octochrome (eight-chamber) and multiprobe (24-chamber) devices. Briefly, labeled probes were air dried on to the device. Probes were rehybridized in standard buffer and applied to the glass slide (which was subdivided to correspond to the hybridization chambers), and FISH continued as above.

### EBR detection and CNE density analysis

The multiple alignments of the chicken, zebra finch, flycatcher, pigeon, and falcon chromosome sequences were obtained using progressiveCactus (Paten et al. 2011) with default parameters. Pairwise synteny blocks were defined using the maf2synteny tool (Kolmogorov et al. 2014) at 100-, 300-, and 500-kb resolution. By using chicken as the reference genome, EBRs were detected and classified using the ad hoc statistical approach described previously (Farré et al. 2016). All well-defined (or flanking oriented PCFs) fusion and fission points were identified from pairwise alignments with the chicken genome. Only the EBRs ≤100 kb were used for the CNE analysis. EBRs <1 kb were extended ±1 kb. For each EBR, we defined two windows upstream of (+1 and +2) and two downstream from (−1 and −2) the same size as the EBR. We calculated the fraction of bases within CNEs in each EBR site and the upstream and downstream windows. Differences in CNE densities were tested for significance using the Kruskall-Wallis test followed by Mann-Whitney *U* test.

### Comparing CNE densities in EBRs and msHSBs

Chicken chromosomes (excluding GGA16, W, and Z) were divided into 1-kb nonoverlapping intervals. Only windows with >50% of their bases with chicken sequence data available were used in this analysis. All intervals were assigned either to msHSBs >1.5 Mb (Farré et al. 2016); to avian EBRs flanking fusions, fissions, and intrachromosomal EBR; and to the intervals found in the rest of the chicken genome. We estimated the average CNE density for each window type and also the distance, in number of 1-kb windows, between each window with the lowest CNE density (0 bp) and the nearest window with the average msHSB CNE density or higher. CNE densities were obtained using BEDtools (v.2.20-1) (Quinlan and Hall 2010). Differences in distances between the two window types in msHSBs and EBRs were tested for significance using the Kruskall-Wallis test followed by Mann-Whitney *U* test.

### Densities of TEs in falcon intrachromosomal EBRs, fusions, and fissions

The TE scaffold coordinates reported by Zhan et al. (2013) were translated to falcon chromosome coordinates using a custom Perl script. The densities of TEs (>100 bp on average in the EBR- or non-EBR-containing nonoverlapping 10-kb genome intervals) were compared for the falcon lineage–specific interchromosomal EBRs, EBRs flanking fusion and fission events, and the rest of the genome as previously described (Elsik et al. 2009; Larkin et al. 2009; Groenen et al. 2012; Farré et al. 2016).

## Data access

The falcon and pigeon chromosome assemblies from this study have been submitted to DDBJ/ENA/GenBank (https://www.ncbi.nlm.nih.gov/genbank/) under the accessions numbers MLQY00000000 and MLQZ00000000, respectively. Visualizations of falcon and pigeon genome assemblies are available from the Evolution Highway comparative chromosome browser (http://eh-demo.ncsa.uiuc.edu/birds) and our UCSC Genome Browser hub (http://sftp.rvc.ac.uk/rvcpaper/birdsHUB/hub.txt).

## Supplementary Material

Supplemental Material

## References

[DAMASGR213660C1] Andersson L, Georges M. 2004 Domestic-animal genomics: deciphering the genetics of complex traits. Nat Rev Genet 5: 202–212. 14970822 10.1038/nrg1294

[DAMASGR213660C2] Baudet C, Lemaitre C, Dias Z, Gautier C, Tannier E, Sagot MF. 2010 Cassis: detection of genomic rearrangement breakpoints. Bioinformatics 26: 1897–1898. 20576622 10.1093/bioinformatics/btq301 PMC2905553

[DAMASGR213660C3] Benson G. 1999 Tandem repeats finder: a program to analyze DNA sequences. Nucleic Acids Res 27: 573–580. 9862982 10.1093/nar/27.2.573 PMC148217

[DAMASGR213660C4] Branco MR, Pombo A. 2006 Intermingling of chromosome territories in interphase suggests role in translocations and transcription-dependent associations. PLoS Biol 4: e138. 16623600 10.1371/journal.pbio.0040138 PMC1440941

[DAMASGR213660C5] Burt DW. 2002 Origin and evolution of avian microchromosomes. Cytogenet Genome Res 96: 97–112. 12438785 10.1159/000063018

[DAMASGR213660C6] Burt DW, Bruley C, Dunn IC, Jones CT, Ramage A, Law AS, Morrice DR, Paton IR, Smith J, Windsor D, 1999 The dynamics of chromosome evolution in birds and mammals. Nature 402: 411–413. 10586880 10.1038/46555

[DAMASGR213660C7] Bush GL, Case SM, Wilson AC, Patton JL. 1977 Rapid speciation and chromosomal evolution in mammals. Proc Natl Acad Sci 74: 3942–3946. 269445 10.1073/pnas.74.9.3942 PMC431793

[DAMASGR213660C8] Dalloul RA, Long JA, Zimin AV, Aslam L, Beal K, Blomberg Le A, Bouffard P, Burt DW, Crasta O, Crooijmans RP, 2010 Multi-platform next-generation sequencing of the domestic turkey (*Meleagris gallopavo*): genome assembly and analysis. PLoS Biol 8: e1000475. 20838655 10.1371/journal.pbio.1000475 PMC2935454

[DAMASGR213660C9] Deakin JE, Ezaz T. 2014 Tracing the evolution of amniote chromosomes. Chromosoma 123: 201–216. 24664317 10.1007/s00412-014-0456-y PMC4031395

[DAMASGR213660C10] Derjusheva S, Kurganova A, Habermann F, Gaginskaya E. 2004 High chromosome conservation detected by comparative chromosome painting in chicken, pigeon and passerine birds. Chromosome Res 12: 715–723. 15505406 10.1023/B:CHRO.0000045779.50641.00

[DAMASGR213660C11] Driscoll CA, Macdonald DW, O'Brien SJ. 2009 From wild animals to domestic pets, an evolutionary view of domestication. Proc Natl Acad Sci 106: 9971–9978. 19528637 10.1073/pnas.0901586106 PMC2702791

[DAMASGR213660C12] Ellegren H, Smeds L, Burri R, Olason PI, Backstrom N, Kawakami T, Kunstner A, Makinen H, Nadachowska-Brzyska K, Qvarnstrom A, 2012 The genomic landscape of species divergence in *Ficedula* flycatchers. Nature 491: 756–760. 23103876 10.1038/nature11584

[DAMASGR213660C13] Elsik CG, Tellam RL, Worley KC. 2009 The genome sequence of a taurine cattle: a window to ruminant biology and evolution. Science 324: 522–528. 19390049 10.1126/science.1169588 PMC2943200

[DAMASGR213660C14] Fang X, Nevo E, Han L, Levanon EY, Zhao J, Avivi A, Larkin D, Jiang X, Feranchuk S, Zhu Y, 2014 Genome-wide adaptive complexes to underground stresses in blind mole rats *Spalax*. Nat Commun 5: 3966. 24892994 10.1038/ncomms4966

[DAMASGR213660C15] Farré M, Narayan J, Slavov GT, Damas J, Auvil L, Li C, Jarvis ED, Burt DW, Griffin DK, Larkin DM. 2016 Novel insights into chromosome evolution in birds, archosaurs, and reptiles. Genome Biol Evol 8: 2442–2451. 27401172 10.1093/gbe/evw166 PMC5010900

[DAMASGR213660C16] Ferguson-Lees J, Christie DA. 2005 Raptors of the world. Princeton University Press, Princeton, NJ.

[DAMASGR213660C17] Fontdevila A, Ruiz A, Ocaña J, Alonso G. 1982 Evolutionary history of *Drosophila buzzatii*. II. How much has chromosomal polymorphism changed in colonization? Evolution 36: 843–851. 10.1111/j.1558-5646.1982.tb05450.x 28568228

[DAMASGR213660C18] Griffin DK, Robertson LBW, Tempest HG, Skinner BM. 2007 The evolution of the avian genome as revealed by comparative molecular cytogenetics. Cytogenet Genome Res 117: 64–77. 17675846 10.1159/000103166

[DAMASGR213660C19] Groenen MAM, Archibald AL, Uenishi H, Tuggle CK, Takeuchi Y, Rothschild MF, Rogel-Gaillard C, Park C, Milan D, Megens H-J, 2012 Analyses of pig genomes provide insight into porcine demography and evolution. Nature 491: 393–398. 23151582 10.1038/nature11622 PMC3566564

[DAMASGR213660C20] Hansmann T, Nanda I, Volobouev V, Yang F, Schartl M, Haaf T, Schmid M. 2009 Cross-species chromosome painting corroborates microchromosome fusion during karyotype evolution of birds. Cytogenet Genome Res 126: 281–304. 20068299 10.1159/000251965

[DAMASGR213660C21] Harris RS. 2007 “Improved pairwise alignment of genomic DNA.” PhD thesis, The Pennsylvania State University, State College, PA.

[DAMASGR213660C22] Hillier L. 2004 Sequence and comparative analysis of the chicken genome provide unique perspectives on vertebrate evolution. Nature 432: 695–716. 15592404 10.1038/nature03154

[DAMASGR213660C23] Hu X, Gao Y, Feng C, Liu Q, Wang X, Du Z, Wang Q, Li N. 2009 Advanced technologies for genomic analysis in farm animals and its application for QTL mapping. Genetica 136: 371–386. 19093212 10.1007/s10709-008-9338-7

[DAMASGR213660C24] Jarvis ED, Mirarab S, Aberer AJ, Li B, Houde P, Li C, Ho SYW, Faircloth BC, Nabholz B, Howard JT, 2014 Whole-genome analyses resolve early branches in the tree of life of modern birds. Science 346: 1320–1331. 25504713 10.1126/science.1253451 PMC4405904

[DAMASGR213660C25] Kasai F, O'Brien PC, Ferguson-Smith MA. 2012 Reassessment of genome size in turtle and crocodile based on chromosome measurement by flow karyotyping: close similarity to chicken. Biol Lett 8: 631–635. 22491763 10.1098/rsbl.2012.0141 PMC3391471

[DAMASGR213660C26] Kasai F, O'Brien PC, Ferguson-Smith MA. 2013 Afrotheria genome; overestimation of genome size and distinct chromosome GC content revealed by flow karyotyping. Genomics 102: 468–471. 24055950 10.1016/j.ygeno.2013.09.002

[DAMASGR213660C27] Kent WJ, Sugnet CW, Furey TS, Roskin KM, Pringle TH, Zahler AM, Haussler D. 2002 The human genome browser at UCSC. Genome Res 12: 996–1006. 12045153 10.1101/gr.229102 PMC186604

[DAMASGR213660C28] Kent WJ, Baertsch R, Hinrichs A, Miller W, Haussler D. 2003 Evolution's cauldron: duplication, deletion, and rearrangement in the mouse and human genomes. Proc Natl Acad Sci 100: 11484–11489. 14500911 10.1073/pnas.1932072100 PMC208784

[DAMASGR213660C29] Kim J, Larkin DM, Cai Q, Asan, Zhang Y, Ge R-L, Auvil L, Capitanu B, Zhang G, Lewin HA, 2013 Reference-assisted chromosome assembly. Proc Natl Acad Sci 110: 1785–1790. 23307812 10.1073/pnas.1220349110 PMC3562798

[DAMASGR213660C30] Kinsella RJ, Kahari A, Haider S, Zamora J, Proctor G, Spudich G, Almeida-King J, Staines D, Derwent P, Kerhornou A, 2011 Ensembl BioMarts: a hub for data retrieval across taxonomic space. Database 2011: bar030. 21785142 10.1093/database/bar030 PMC3170168

[DAMASGR213660C31] Koepfli K-P, Paten B, Scientists tGKCo, O'Brien SJ. 2015 The Genome 10K Project: a way forward. Ann Rev Anim Biosci 3: 57–111. 25689317 10.1146/annurev-animal-090414-014900 PMC5837290

[DAMASGR213660C32] Kolmogorov M, Raney B, Paten B, Pham S. 2014 Ragout-a reference-assisted assembly tool for bacterial genomes. Bioinformatics 30: i302–309. 24931998 10.1093/bioinformatics/btu280 PMC4058940

[DAMASGR213660C33] Larkin DM, Prokhorovich MA, Astakhova NM, Zhdanova NS. 2006 Comparative mapping of mink chromosome 8p: in situ hybridization of seven cattle BAC clones. Anim Genet 37: 429–430. 16879369 10.1111/j.1365-2052.2006.01491.x

[DAMASGR213660C34] Larkin DM, Pape G, Donthu R, Auvil L, Welge M, Lewin HA. 2009 Breakpoint regions and homologous synteny blocks in chromosomes have different evolutionary histories. Genome Res 19: 770–777. 19342477 10.1101/gr.086546.108 PMC2675965

[DAMASGR213660C35] Larkin DM, Daetwyler HD, Hernandez AG, Wright CL, Hetrick LA, Boucek L, Bachman SL, Band MR, Akraiko TV, Cohen-Zinder M, 2012 Whole-genome resequencing of two elite sires for the detection of haplotypes under selection in dairy cattle. Proc Natl Acad Sci 109: 7693–7698. 22529356 10.1073/pnas.1114546109 PMC3356612

[DAMASGR213660C36] Lewin HA, Larkin DM, Pontius J, O'Brien SJ. 2009 Every genome sequence needs a good map. Genome Res 19: 1925–1928. 19596977 10.1101/gr.094557.109 PMC2775595

[DAMASGR213660C37] Lindblad-Toh K, Garber M, Zuk O, Lin MF, Parker BJ, Washietl S, Kheradpour P, Ernst J, Jordan G, Mauceli E, 2011 A high-resolution map of human evolutionary constraint using 29 mammals. Nature 478: 476–482. 21993624 10.1038/nature10530 PMC3207357

[DAMASGR213660C38] Loh W-Y. 2011 Classification and regression trees. WIREs Data Mining Knowl Discov 1: 14–23.

[DAMASGR213660C39] Mak ACY, Lai YYY, Lam ET, Kwok T-P, Leung AKY, Poon A, Mostovoy Y, Hastie AR, Stedman W, Anantharaman T, 2016 Genome-wide structural variation detection by genome mapping on nanochannel arrays. Genetics 202: 351–362. 26510793 10.1534/genetics.115.183483 PMC4701098

[DAMASGR213660C40] Makunin AI, Kichigin IG, Larkin DM, O'Brien PC, Ferguson-Smith MA, Yang F, Proskuryakova AA, Vorobieva NV, Chernyaeva EN, O'Brien SJ, 2016 Contrasting origin of B chromosomes in two cervids (Siberian roe deer and grey brocket deer) unravelled by chromosome-specific DNA sequencing. BMC Genomics 17: 618. 27516089 10.1186/s12864-016-2933-6 PMC4982142

[DAMASGR213660C41] Meyerson M, Gabriel S, Getz G. 2010 Advances in understanding cancer genomes through second-generation sequencing. Nat Rev Genet 11: 685–696. 20847746 10.1038/nrg2841

[DAMASGR213660C42] Modi WS, Romanov M, Green ED, Ryder O. 2009 Molecular cytogenetics of the California condor: evolutionary and conservation implications. Cytogenet Genome Res 127: 26–32. 20051671 10.1159/000272458 PMC2842169

[DAMASGR213660C43] Morgulis A, Gertz EM, Schaffer AA, Agarwala R. 2006 A fast and symmetric DUST implementation to mask low-complexity DNA sequences. J Comput Biol 13: 1028–1040. 16796549 10.1089/cmb.2006.13.1028

[DAMASGR213660C44] Murphy WJ, Larkin DM, Everts-van der Wind A, Bourque G, Tesler G, Auvil L, Beever JE, Chowdhary BP, Galibert F, Gatzke L, 2005 Dynamics of mammalian chromosome evolution inferred from multispecies comparative maps. Science 309: 613–617. 16040707 10.1126/science.1111387

[DAMASGR213660C45] Nishida C, Ishijima J, Kosaka A, Tanabe H, Habermann FA, Griffin DK, Matsuda Y. 2008 Characterization of chromosome structures of Falconinae (Falconidae, Falconiformes, Aves) by chromosome painting and delineation of chromosome rearrangements during their differentiation. Chromosome Res 16: 171–181. 18293111 10.1007/s10577-007-1210-6

[DAMASGR213660C46] Paten B, Earl D, Nguyen N, Diekhans M, Zerbino D, Haussler D. 2011 Cactus: algorithms for genome multiple sequence alignment. Genome Res 21: 1512–1528. 21665927 10.1101/gr.123356.111 PMC3166836

[DAMASGR213660C47] Price TD. 2002 Domesticated birds as a model for the genetics of speciation by sexual selection. Genetica 116: 311–327. 12555787

[DAMASGR213660C48] Putnam NH, O'Connell BL, Stites JC, Rice BJ, Blanchette M, Calef R, Troll CJ, Fields A, Hartley PD, Sugnet CW, 2016 Chromosome-scale shotgun assembly using an in vitro method for long-range linkage. Genome Res 26: 342–350. 26848124 10.1101/gr.193474.115 PMC4772016

[DAMASGR213660C49] Quinlan AR, Hall IM. 2010 BEDTools: a flexible suite of utilities for comparing genomic features. Bioinformatics 26: 841–842. 20110278 10.1093/bioinformatics/btq033 PMC2832824

[DAMASGR213660C50] Rhoads A, Au KF. 2015 PacBio sequencing and its applications. Genomics Proteomics Bioinformatics 13: 278–289. 26542840 10.1016/j.gpb.2015.08.002 PMC4678779

[DAMASGR213660C51] Romanov MN, Dodgson JB, Gonser RA, Tuttle EM. 2011 Comparative BAC-based mapping in the white-throated sparrow, a novel behavioral genomics model, using interspecies overgo hybridization. BMC Res Notes 4: 211. 21693052 10.1186/1756-0500-4-211 PMC3155834

[DAMASGR213660C52] Romanov MN, Farré M, Lithgow PE, Fowler KE, Skinner BM, O'Connor R, Fonseka G, Backström N, Matsuda Y, Nishida C, 2014 Reconstruction of gross avian genome structure, organization and evolution suggests that the chicken lineage most closely resembles the dinosaur avian ancestor. BMC Genomics 15: 1–18. 25496766 10.1186/1471-2164-15-1060 PMC4362836

[DAMASGR213660C53] Rosenbloom KR, Armstrong J, Barber GP, Casper J, Clawson H, Diekhans M, Dreszer TR, Fujita PA, Guruvadoo L, Haeussler M, 2015 The UCSC Genome Browser database: 2015 update. Nucleic Acids Res 43: D670–D681. 25428374 10.1093/nar/gku1177 PMC4383971

[DAMASGR213660C54] Schmid M, Smith J, Burt DW, Aken BL, Antin PB, Archibald AL, Ashwell C, Blackshear PJ, Boschiero C, Brown CT, 2015 Third report on chicken genes and chromosomes 2015. Cytogenet Genome Res 145: 78–179. 26282327 10.1159/000430927 PMC5120589

[DAMASGR213660C55] Schneider VA, Chen HC, Clausen C, Meric PA, Zhou Z, Bouk N, Husain N, Maglott DR, Church DM. 2013 Clone DB: an integrated NCBI resource for clone-associated data. Nucleic Acids Res 41: D1070–D1078. 23193260 10.1093/nar/gks1164 PMC3531087

[DAMASGR213660C56] Shapiro MD, Kronenberg Z, Li C, Domyan ET, Pan H, Campbell M, Tan H, Huff CD, Hu H, Vickrey AI, 2013 Genomic diversity and evolution of the head crest in the rock pigeon. Science 339: 1063–1067. 23371554 10.1126/science.1230422 PMC3778192

[DAMASGR213660C57] Siepel A, Bejerano G, Pedersen JS, Hinrichs AS, Hou M, Rosenbloom K, Clawson H, Spieth J, Hillier LW, Richards S, 2005 Evolutionarily conserved elements in vertebrate, insect, worm, and yeast genomes. Genome Res 15: 1034–2050. 16024819 10.1101/gr.3715005 PMC1182216

[DAMASGR213660C58] Skinner BM, Griffin DK. 2012 Intrachromosomal rearrangements in avian genome evolution: evidence for regions prone to breakpoints. Heredity 108: 37–41. 22045382 10.1038/hdy.2011.99 PMC3238122

[DAMASGR213660C59] Stringham SA, Mulroy EE, Xing J, Record D, Guernsey MW, Aldenhoven JT, Osborne EJ, Shapiro MD. 2012 Divergence, convergence, and the ancestry of feral populations in the domestic rock pigeon. Curr Biol 22: 302–308. 22264611 10.1016/j.cub.2011.12.045 PMC3288640

[DAMASGR213660C60] Tamazian G, Dobrynin P, Krasheninnikova K, Komissarov A, Koepfli KP, O'Brien SJ. 2016 Chromosomer: a reference-based genome arrangement tool for producing draft chromosome sequences. GigaScience 5: 38. 27549770 10.1186/s13742-016-0141-6 PMC4994284

[DAMASGR213660C61] Teague B, Waterman MS, Goldstein S, Potamousis K, Zhou S, Reslewic S, Sarkar D, Valouev A, Churas C, Kidd JM, 2010 High-resolution human genome structure by single-molecule analysis. Proc Natl Acad Sci 107: 10848–10853. 20534489 10.1073/pnas.0914638107 PMC2890719

[DAMASGR213660C62] R Core Team. 2015 R: a language and environment for statistical computing. R Foundation for Statistical Computing, Vienna, Austria http://www.R-project.org/.

[DAMASGR213660C64] Tucker V, Cade T, Tucker A. 1998 Diving speeds and angles of a gyrfalcon (*Falco rusticolus*). J Exp Biol 201: 2061–2070. 9622578 10.1242/jeb.201.13.2061

[DAMASGR213660C65] Untergasser A, Cutcutache I, Koressaar T, Ye J, Faircloth BC, Remm M, Rozen SG. 2012 Primer3-new capabilities and interfaces. Nucleic Acids Res 40: e115. 22730293 10.1093/nar/gks596 PMC3424584

[DAMASGR213660C66] Warren WC, Clayton DF, Ellegren H, Arnold AP, Hillier LW, Kunstner A, Searle S, White S, Vilella AJ, Fairley S, 2010 The genome of a songbird. Nature 464: 757–762. 20360741 10.1038/nature08819 PMC3187626

[DAMASGR213660C67] Williams G. 2011 Data mining with Rattle and R: the art of excavating data for knowledge discovery. Springer Science & Business Media, New York.

[DAMASGR213660C68] Zhan X, Pan S, Wang J, Dixon A, He J, Muller MG, Ni P, Hu L, Liu Y, Hou H, 2013 Peregrine and saker falcon genome sequences provide insights into evolution of a predatory lifestyle. Nat Genet 45: 563–566. 23525076 10.1038/ng.2588

[DAMASGR213660C69] Zhang G, Li B, Li C, Gilbert MT, Jarvis ED, Wang J, Avian Genome C. 2014a Comparative genomic data of the Avian Phylogenomics Project. GigaScience 3: 26. 25671091 10.1186/2047-217X-3-26 PMC4322804

[DAMASGR213660C70] Zhang G, Li C, Li Q, Li B, Larkin DM, Lee C, Storz JF, Antunes A, Greenwold MJ, Meredith RW, 2014b Comparative genomics reveals insights into avian genome evolution and adaptation. Science 346: 1311–1320. 25504712 10.1126/science.1251385 PMC4390078

