# Supplementary material for: Upgrading short-read animal genome assemblies to chromosome level using comparative genomics and a universal probe set
Source: Genome Res. 2017 May;27(5):875–84. doi: 10.1101/gr.213660.116 (PMC5411781; doi:10.1101/gr.213660.116)
Supplement: Supplemental Material [file supp_27_5_875__index.html]

Upgrading short-read animal genome assemblies to chromosome level using comparative genomics and a universal probe set — Supplemental Material 

# Upgrading short-read animal genome assemblies to chromosome level using comparative genomics and a universal probe set

## Supplemental Material

- Supplemental\_Fig\_S4.pdf
- Supplemental\_Fig\_S5.pdf
- Supplemental\_Table\_S5.xlsx
- Supplemental\_Table\_S6.xlsx
- Supplemental\_Table\_S7.xlsx
- Supplemental\_Table\_S10.xlsx
- Supplemental\_Material.doc
