# Supplementary material for: Upgrading short-read animal genome assemblies to chromosome level using comparative genomics and a universal probe set
Source: Genome Res. 2017 May;27(5):875–84. doi: 10.1101/gr.213660.116 (PMC5411781; doi:10.1101/gr.213660.116)
Supplement: Supplemental Material [file supp_gr.213660.116_Supplemental_Fig_S4.pdf]

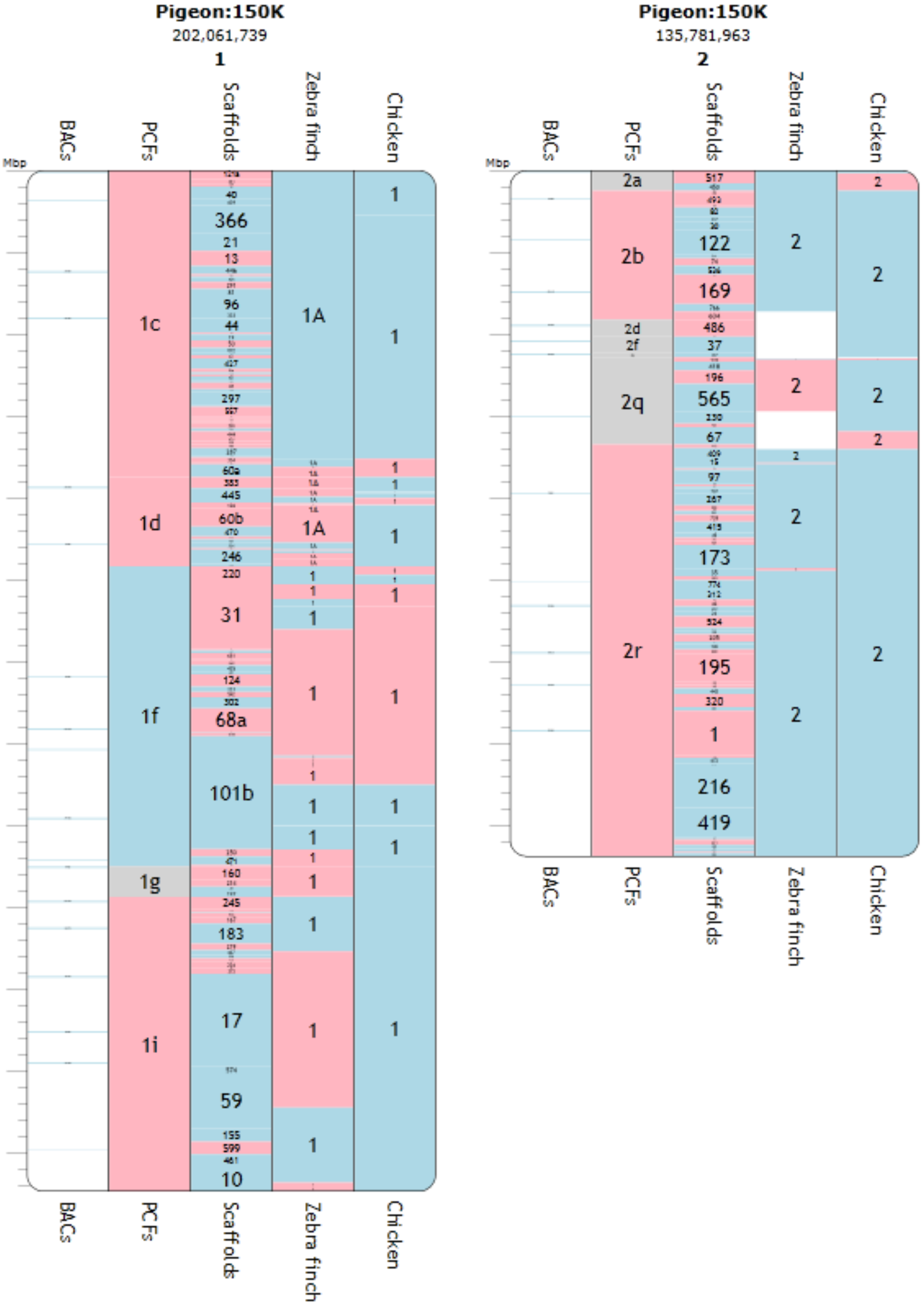
Pigeon:150K
202,061,739
1
BACs
PCFs
Scaffolds
Zebra finch
Chicken
Mbp
1c
1d
1f
1g
1i
40
366
21
13
96
44
427
297
60a
445
60b
470
246
220
31
124
302
68a
101b
160
245
183
17
59
155
599
461
10
1A
1A
1
Pigeon:150K
135,781,963
2
2a
2b
2d
2f
2q
2r
517
493
122
169
486
37
196
565
230
67
97
267
415
173
524
195
320
1
216
419
2

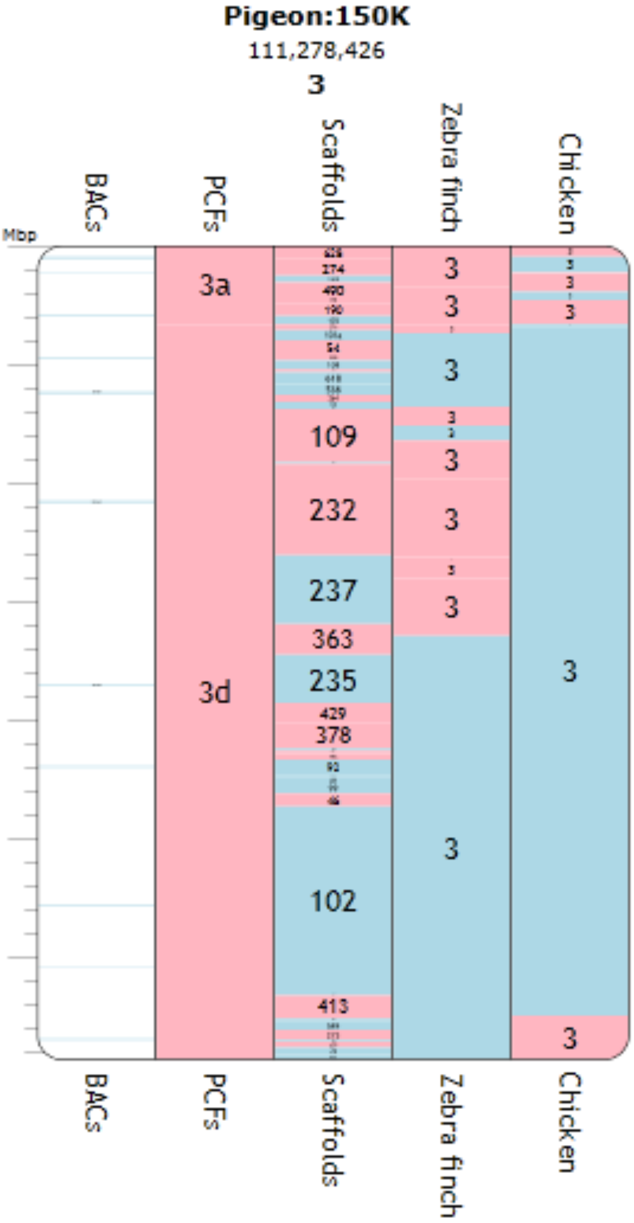
Pigeon:150K
111,278,426
3
BACs
PCFs
Scaffolds
Zebra finch
Chicken
Mbp
3a
3d
109
232
237
363
235
429
378
102
413
3
3

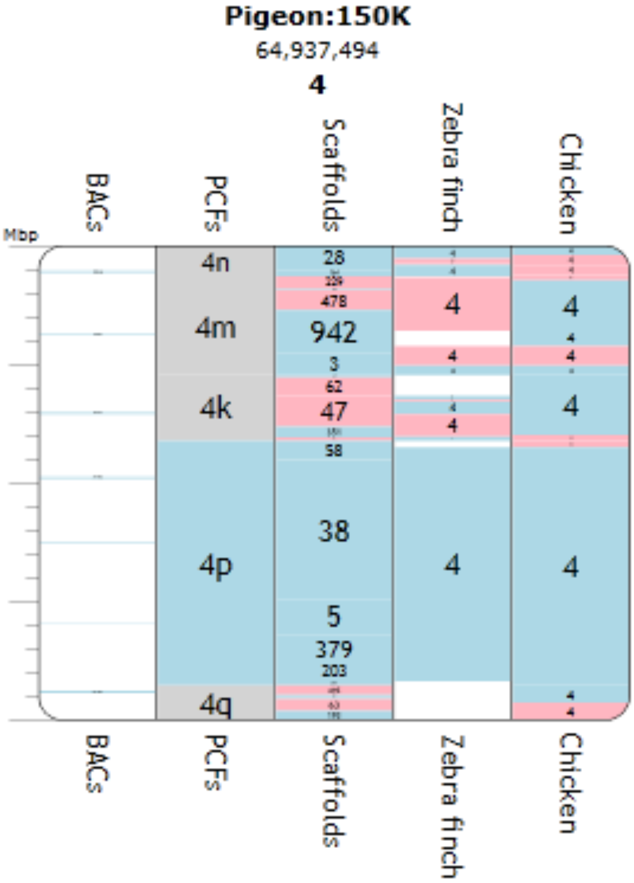
Pigeon:150K
64,937,494
4
BACs
PCFs
Scaffolds
Zebra finch
Chicken
Mbp
4n
4m
4k
4p
4q
28
478
942
3
62
47
58
38
5
379
203
4
4

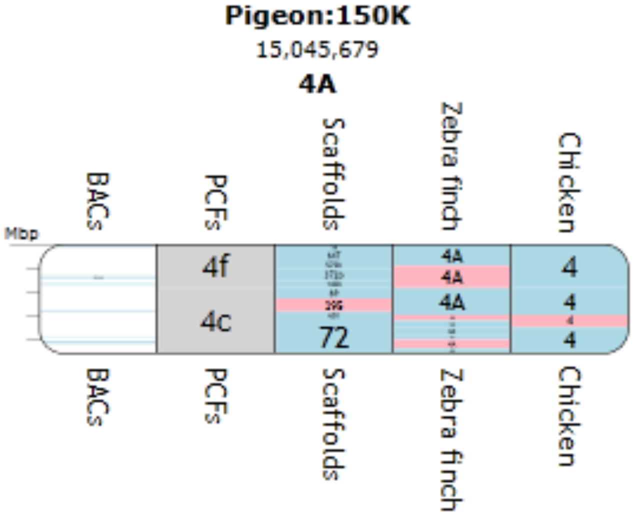
Pigeon:150K
15,045,679
4A
BACs
PCFs
Scaffolds
Zebra finch
Chicken
Mbp
4f
4c
72
4A
4A
4A
4
4
4

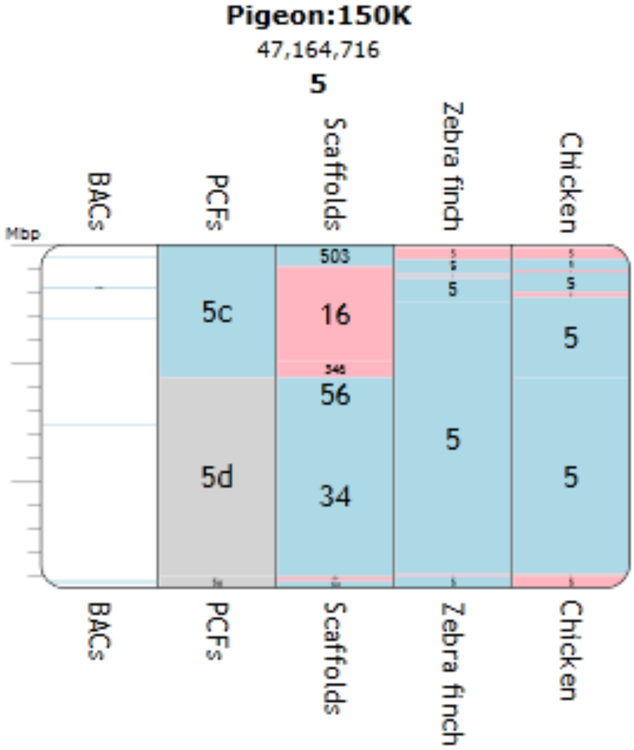
Pigeon:150K
47,164,716
5
BACs
PCFs
Scaffolds
Zebra finch
Chicken
Mbp
5c
5d
503
16
56
34
5
5
5
5

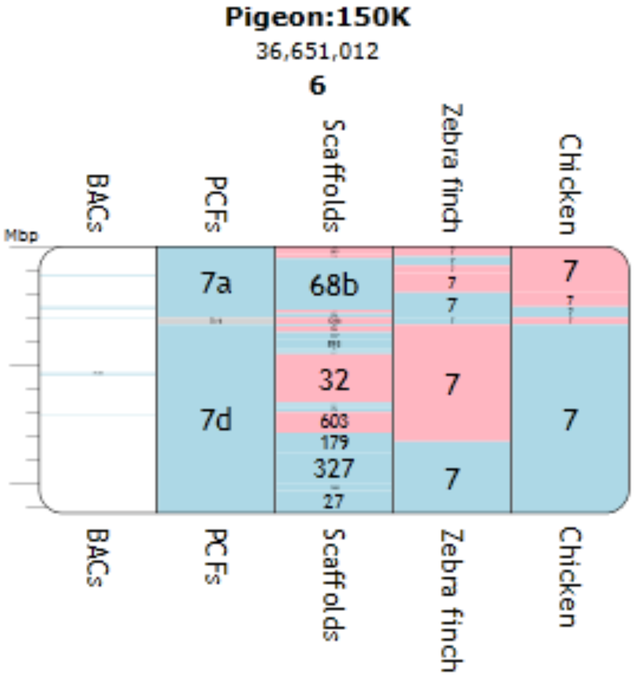
Pigeon:150K
36,651,012
6
BACs
PCFs
Scaffolds
Zebra finch
Chicken
Mbp
7a
7d
68b
32
603
179
327
27
7
7
7
7
7
7

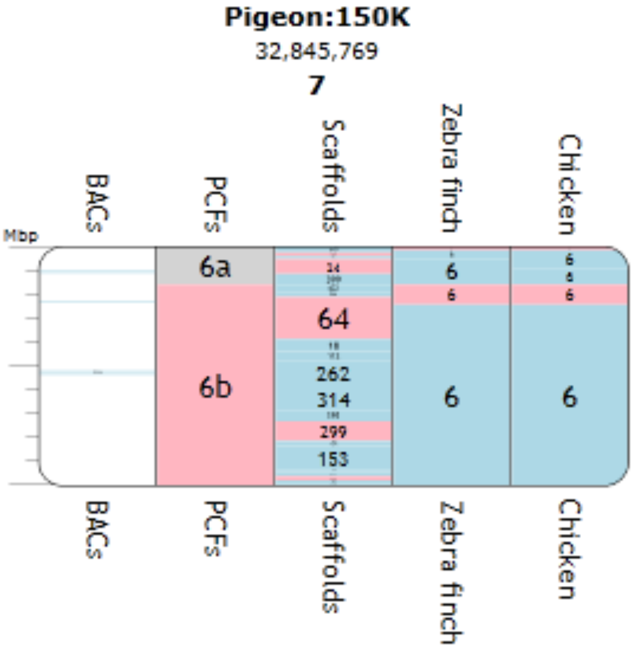
Pigeon:150K
32,845,769
7
BACs
PCFs
Scaffolds
Zebra finch
Chicken
Mbp
6a
6b
34
64
262
314
299
153
6
6
6
6
6
6

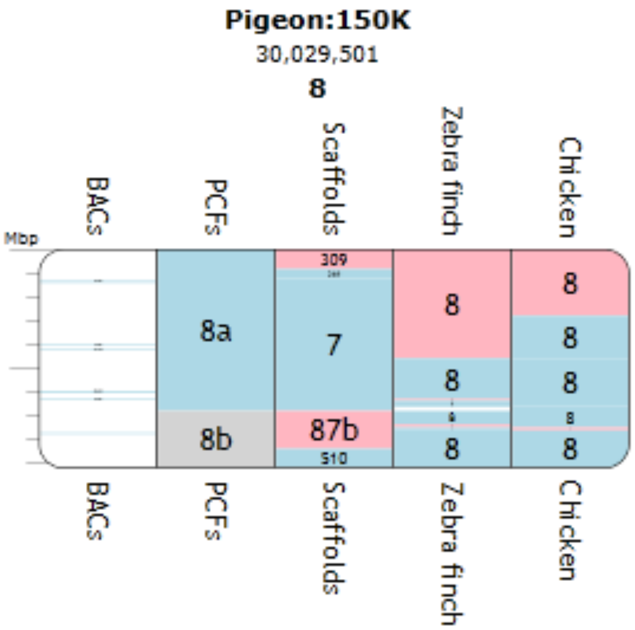
Pigeon:150K
30,029,501
8
BACs
PCFs
Scaffolds
Zebra finch
Chicken
Mbp
8a
8b
309
7
87b
510
8
8
8
8
8
8
8
BACs
PCFs
Scaffolds
Zebra finch
Chicken

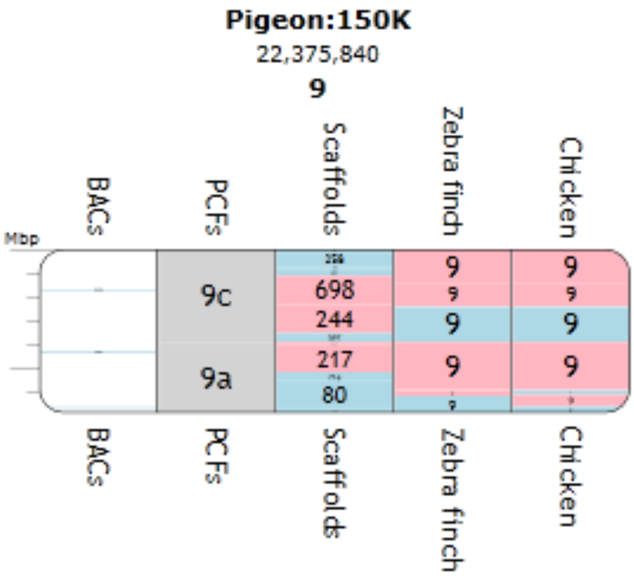
Pigeon:150K
22,375,840
9
BACs
PCFs
Scaffolds
Zebra finch
Chicken
Mbp
9c
9a
698
244
217
80
9
9
9
9
9
9
BACs
PCFs
Scaffolds
Zebra finch
Chicken

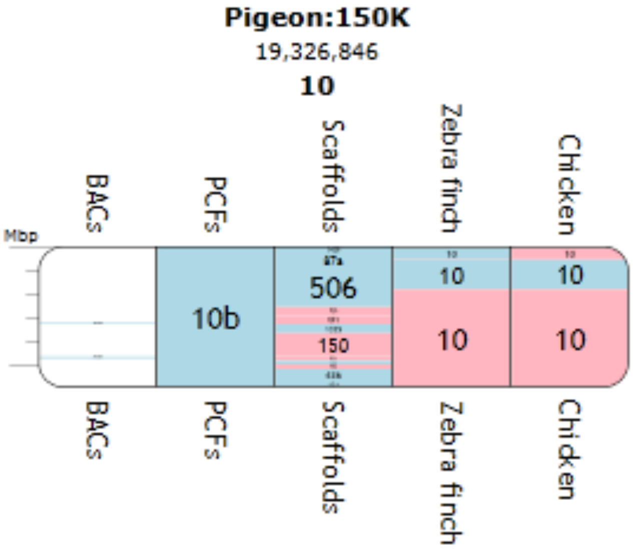
Pigeon:150K
19,326,846
10
Mbp
BACs
PCFs
Scaffolds
Zebra finch
Chicken
10b
506
150
10
10
10
10

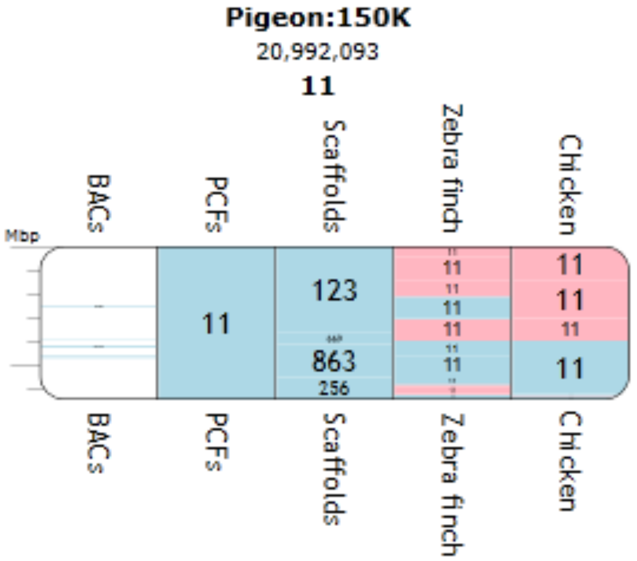
Pigeon:150K
20,992,093
11
Mbp
BACs
PCFs
Scaffolds
Zebra finch
Chicken
11
123
863
256
11
11
11
11
11
11
11
11
11

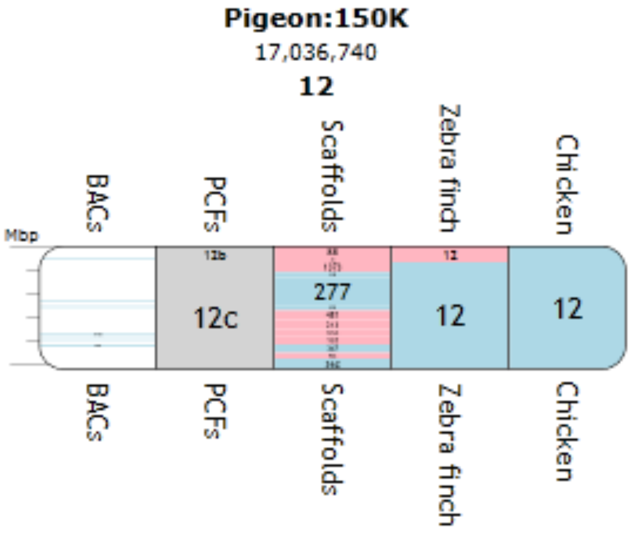
Pigeon:150K
17,036,740
12
BACs
PCFs
Scaffolds
Zebra finch
Chicken
Mbp
12b
277
12c
12
12

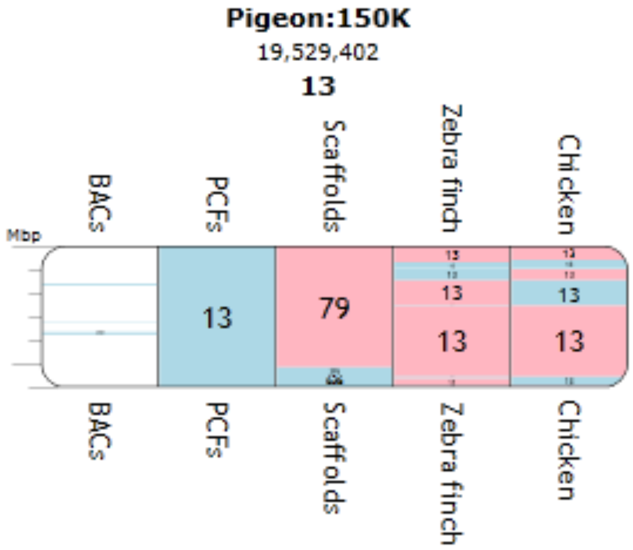
Pigeon:150K
19,529,402
13
BACs
PCFs
Scaffolds
Zebra finch
Chicken
Mbp
13
79
13
13
13
13

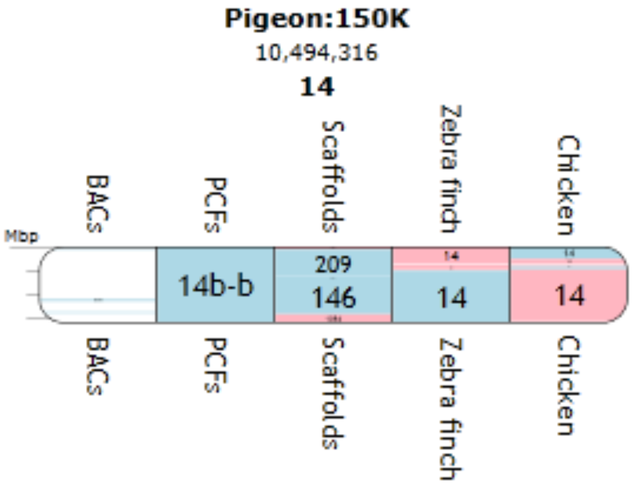
Pigeon:150K
10,494,316
14
BACs
PCFs
Scaffolds
Zebra finch
Chicken
Mbp
14b-b
209
146
14
14

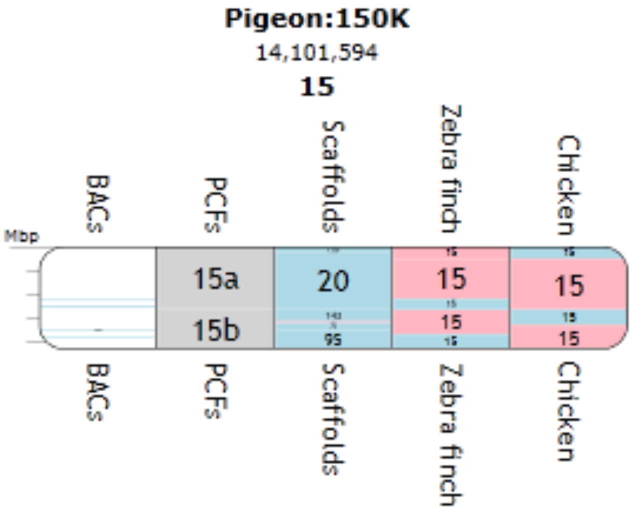
Pigeon:150K
14,101,594
15
BACs
PCFs
Scaffolds
Zebra finch
Chicken
Mbp
15a
15b
20
95
15
15
15
15

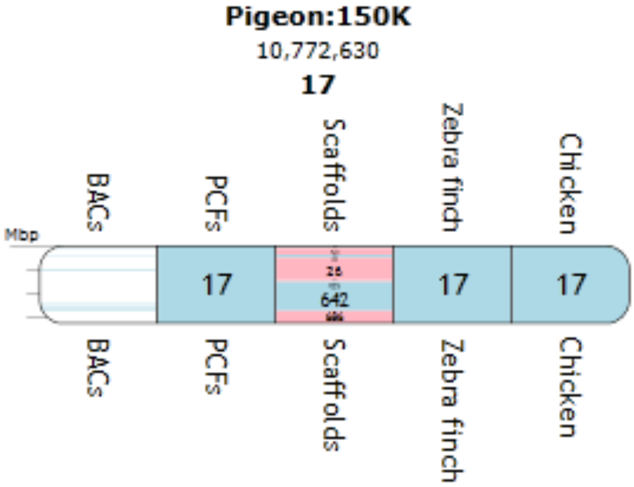
Pigeon:150K
10,772,630
17
Mbp
BACs
PCFs
Scaffolds
Zebra finch
Chicken
17
26
642
17
17

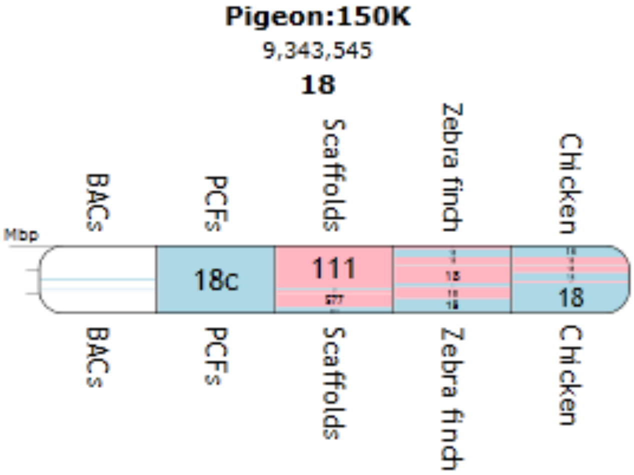
Pigeon:150K
9,343,545
18
Mbp
BACs
PCFs
Scaffolds
Zebra finch
Chicken
18c
111
18

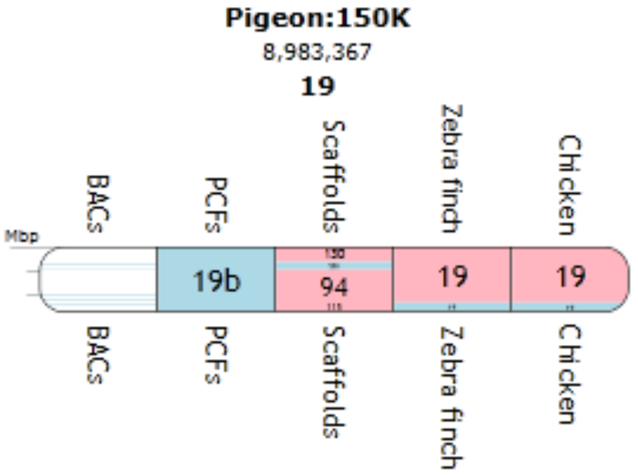
Pigeon:150K
8,983,367
19
Mbp
BACs
PCFs
Scaffolds
Zebra finch
Chicken
19b
94
19
19
BACs
PCFs
Scaffolds
Zebra finch
Chicken

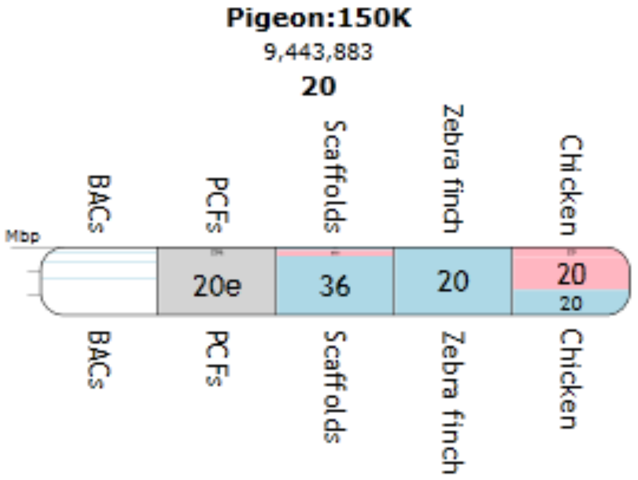
Pigeon:150K
9,443,883
20
Mbp
BACs
PCFs
Scaffolds
Zebra finch
Chicken
20e
36
20
20
20
BACs
PCFs
Scaffolds
Zebra finch
Chicken

Pigeon:150K
5,814,606
21

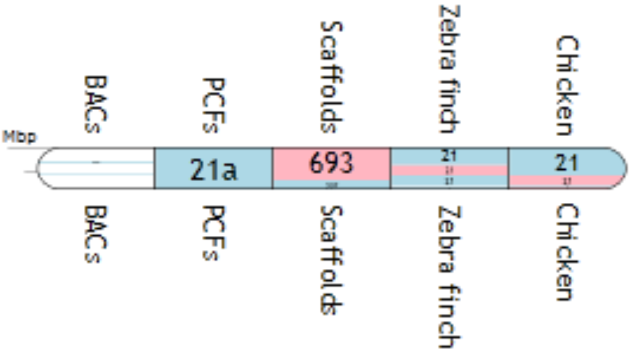

Pigeon:150K
3,913,090
22

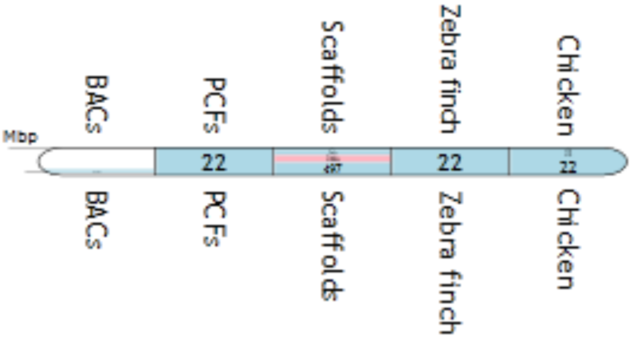

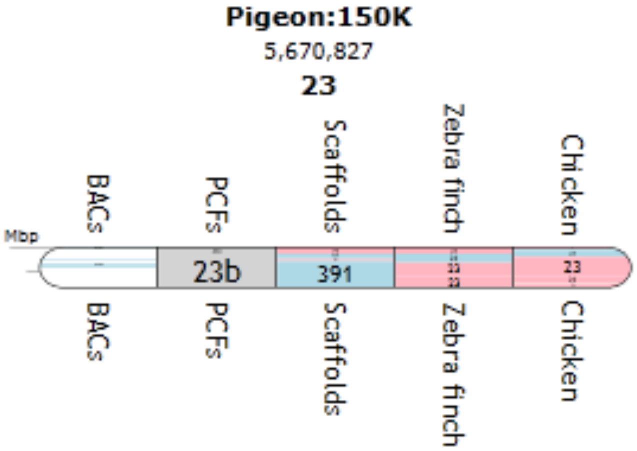
Pigeon:150K
5,670,827
23
BACs
PCFs
Scaffolds
Zebra finch
Chicken
Mbp
23b
391
23
BACs
PCFs
Scaffolds
Zebra finch
Chicken

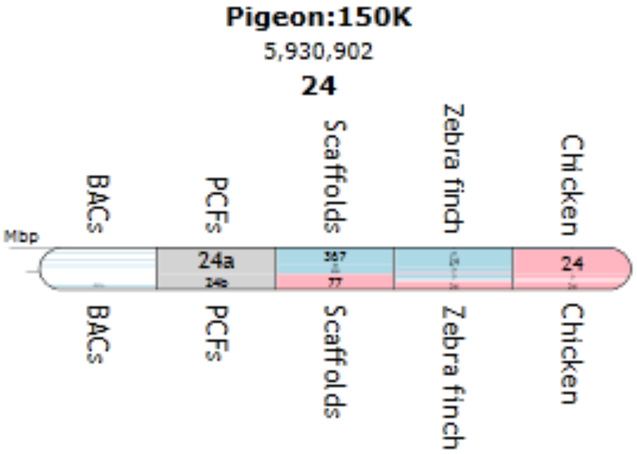
Pigeon:150K
5,930,902
24
BACs
PCFs
Scaffolds
Zebra finch
Chicken
Mbp
24a
24
BACs
PCFs
Scaffolds
Zebra finch
Chicken

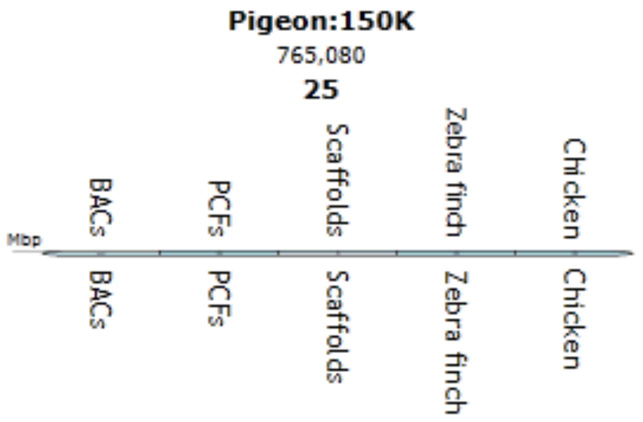
Pigeon:150K
765,080
25
Mbp
BACs
PCFs
Scaffolds
Zebra finch
Chicken
BACs
PCFs
Scaffolds
Zebra finch
Chicken

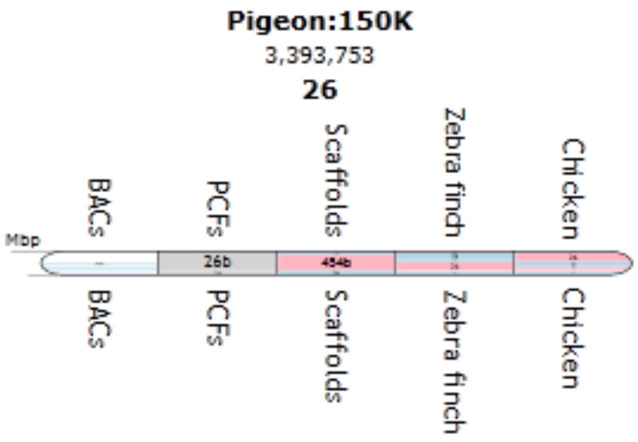
Pigeon:150K
3,393,753
26
Mbp
BACs
PCFs
Scaffolds
Zebra finch
Chicken
26b
BACs
PCFs
Scaffolds
Zebra finch
Chicken

Pigeon:150K
4,918,377
27

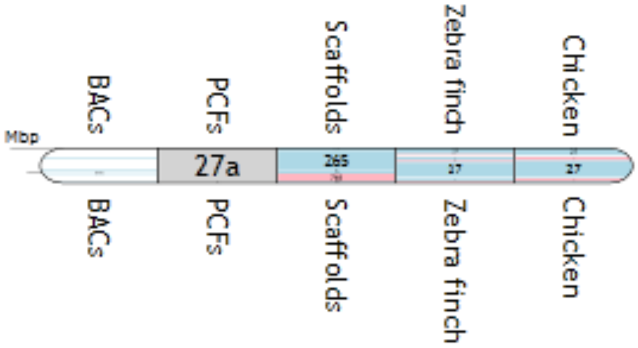

Pigeon:150K
1,542,383
28

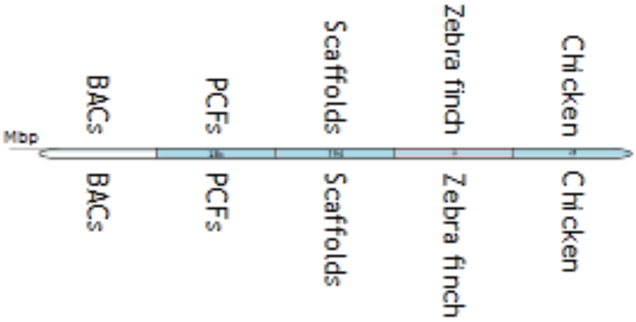

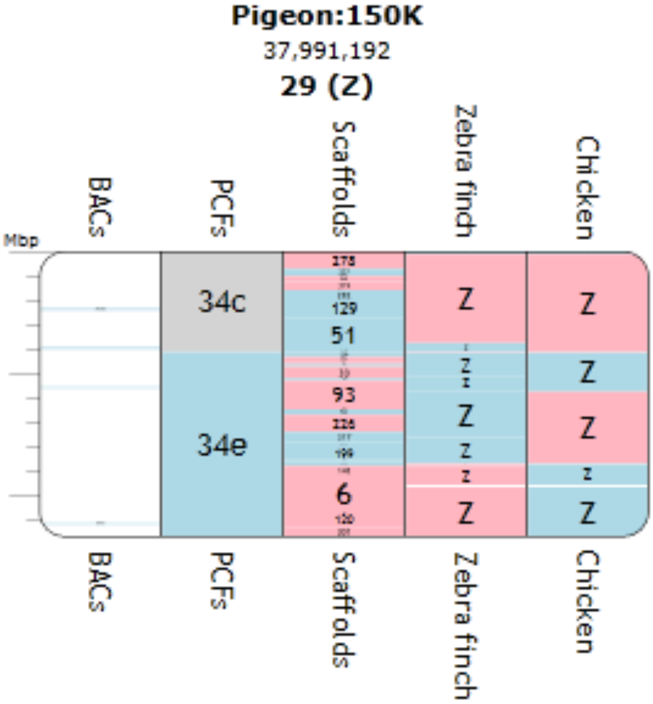
Pigeon:150K
37,991,192
29 (Z)
BACs
PCFs
Scaffolds
Zebra finch
Chicken
Mbp
34c
34e
278
129
51
93
226
199
6
120
Z

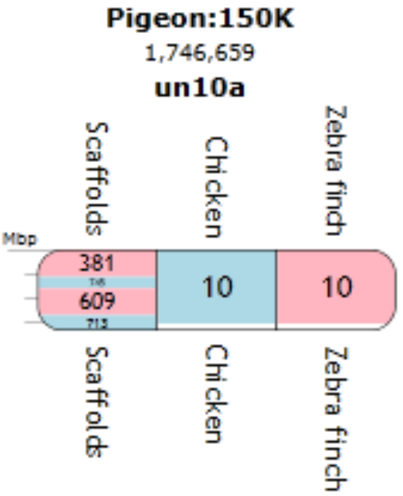
Pigeon:150K
1,746,659
un10a
Scaffolds
Chicken
Zebra finch
Mbp
381
609
10
10
Scaffolds
Chicken
Zebra finch

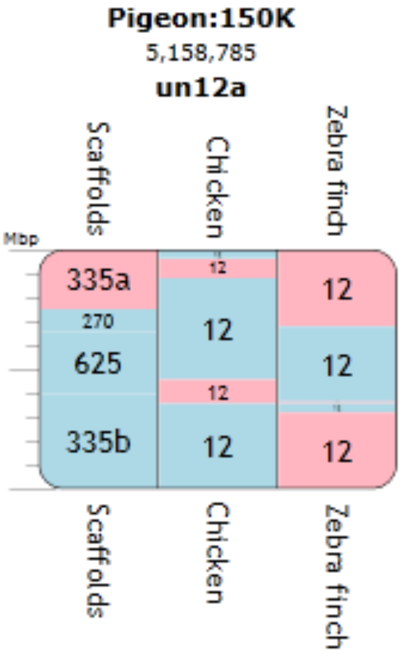
Pigeon:150K
5,158,785
un12a
Scaffolds
Chicken
Zebra finch
Mbp
335a
270
625
335b
12
12
12
12
12
12
12
Scaffolds
Chicken
Zebra finch

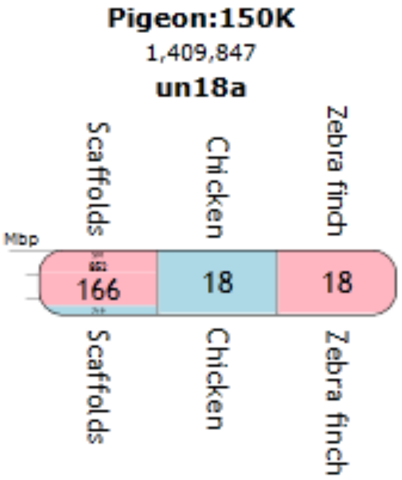
Pigeon:150K
1,409,847
un18a
Scaffolds
Chicken
Zebra finch
Mbp
166
18
18
Scaffolds
Chicken
Zebra finch

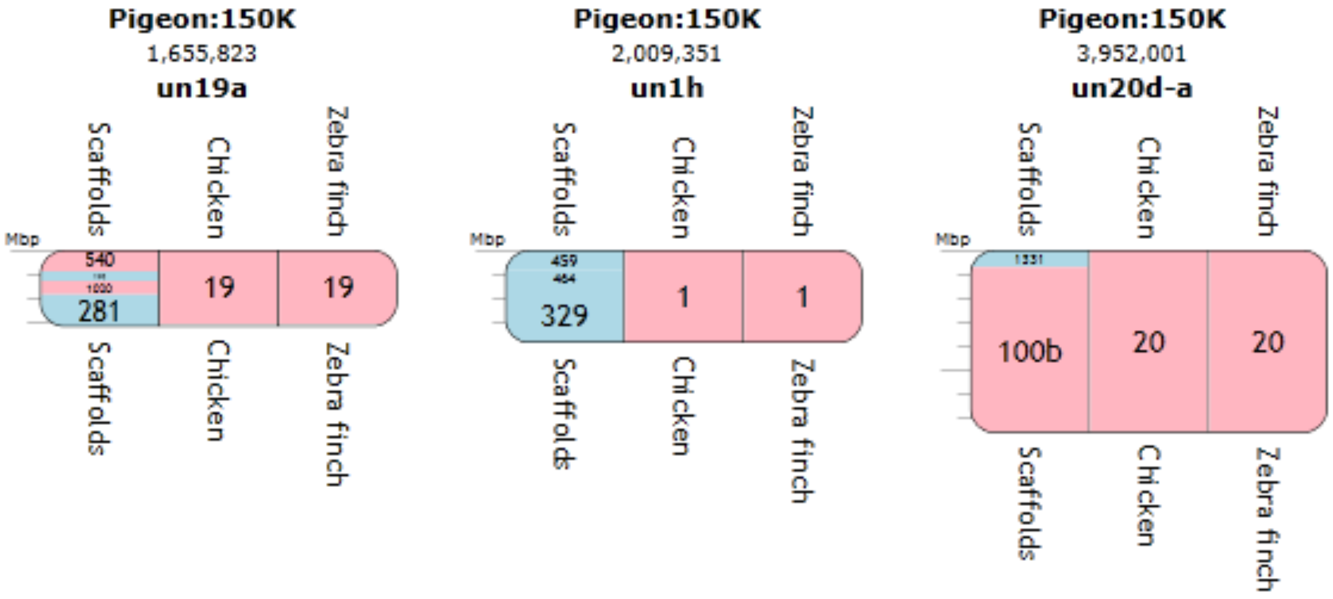
Pigeon:150K
1,655,823
un19a
Scaffolds
Chicken
Zebra finch
Mbp
540
1000
281
19
19
Scaffolds
Chicken
Zebra finch
Pigeon:150K
2,009,351
un1h
Scaffolds
Chicken
Zebra finch
Mbp
459
464
329
1
1
Scaffolds
Chicken
Zebra finch
Pigeon:150K
3,952,001
un20d-a
Scaffolds
Chicken
Zebra finch
Mbp
1331
100b
20
20
Scaffolds
Chicken
Zebra finch

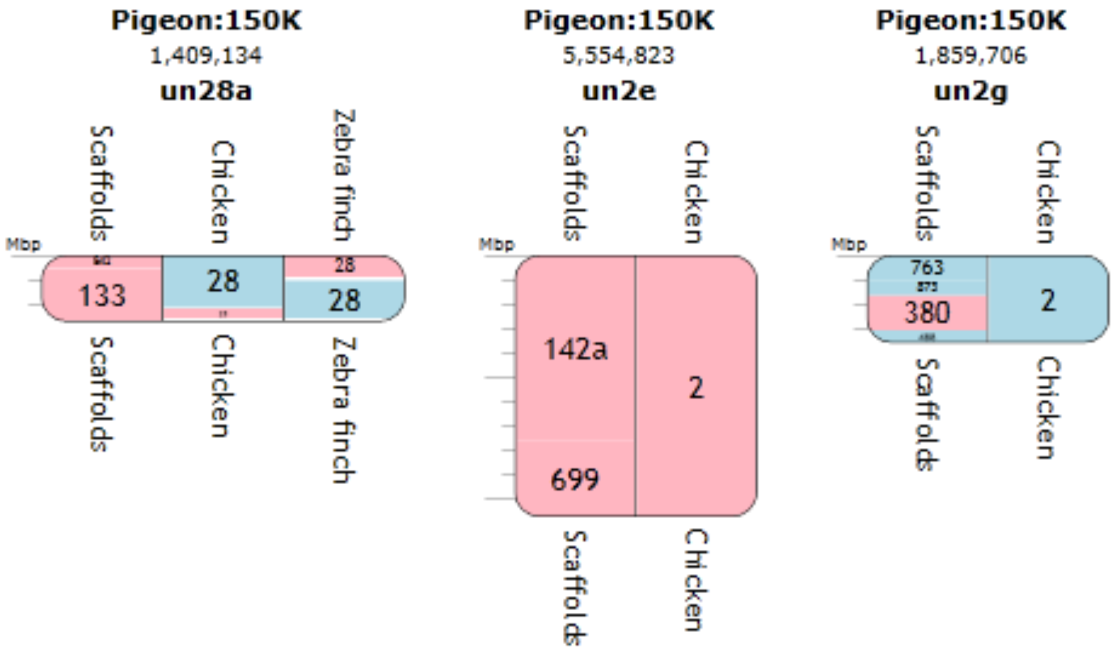
Pigeon:150K
1,409,134
un28a
Scaffolds
Chicken
Zebra finch
Mbp
133
28
28
28
Pigeon:150K
5,554,823
un2e
Scaffolds
Chicken
Mbp
142a
2
699
Pigeon:150K
1,859,706
un2g
Scaffolds
Chicken
Mbp
763
380
2

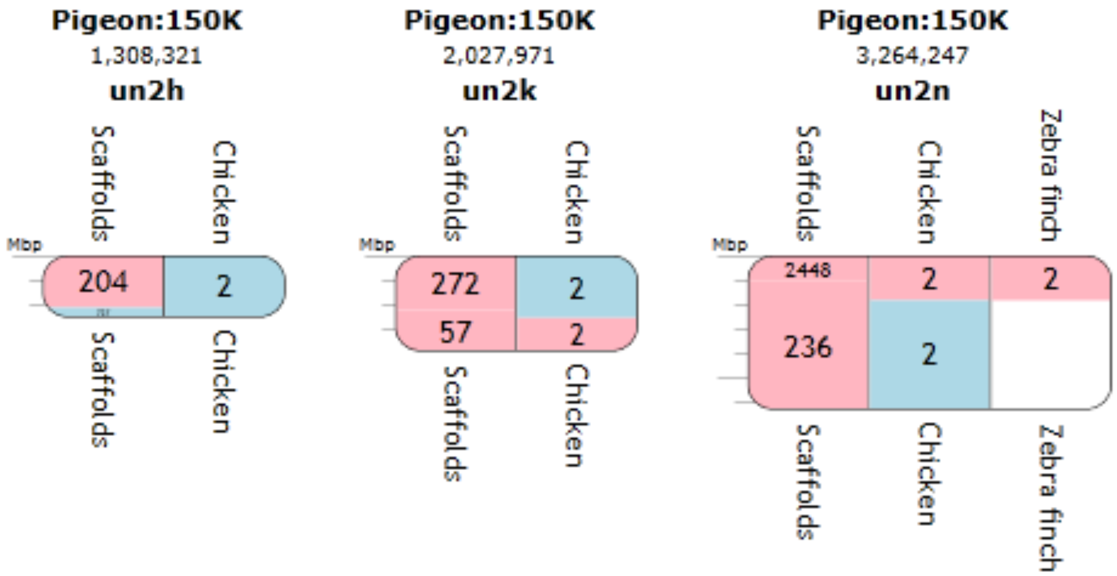
Pigeon:150K
1,308,321
un2h
Scaffolds
Chicken
Mbp
204
2
Scaffolds
Chicken
Pigeon:150K
2,027,971
un2k
Scaffolds
Chicken
Mbp
272
2
57
2
Scaffolds
Chicken
Pigeon:150K
3,264,247
un2n
Scaffolds
Chicken
Zebra finch
Mbp
2448
2
2
236
2
Scaffolds
Chicken
Zebra finch

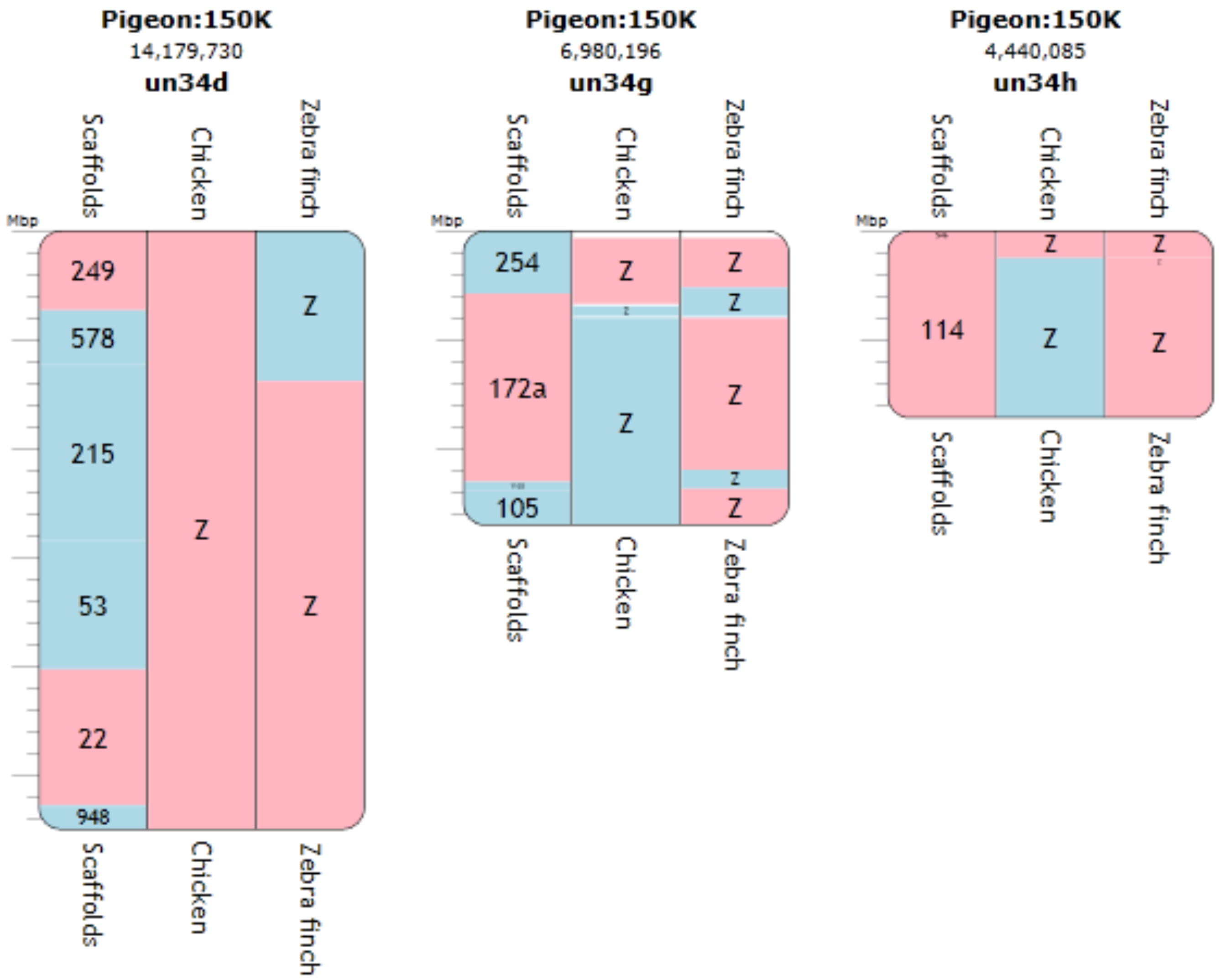
Pigeon:150K
14,179,730
un34d
Scaffolds
Chicken
Zebra finch
Mbp
249
578
215
53
22
948
Z
Z
Z
Pigeon:150K
6,980,196
un34g
254
172a
105
Z
Z
Z
Z
Z
Z
Z
Pigeon:150K
4,440,085
un34h
114
Z
Z
Z
Z

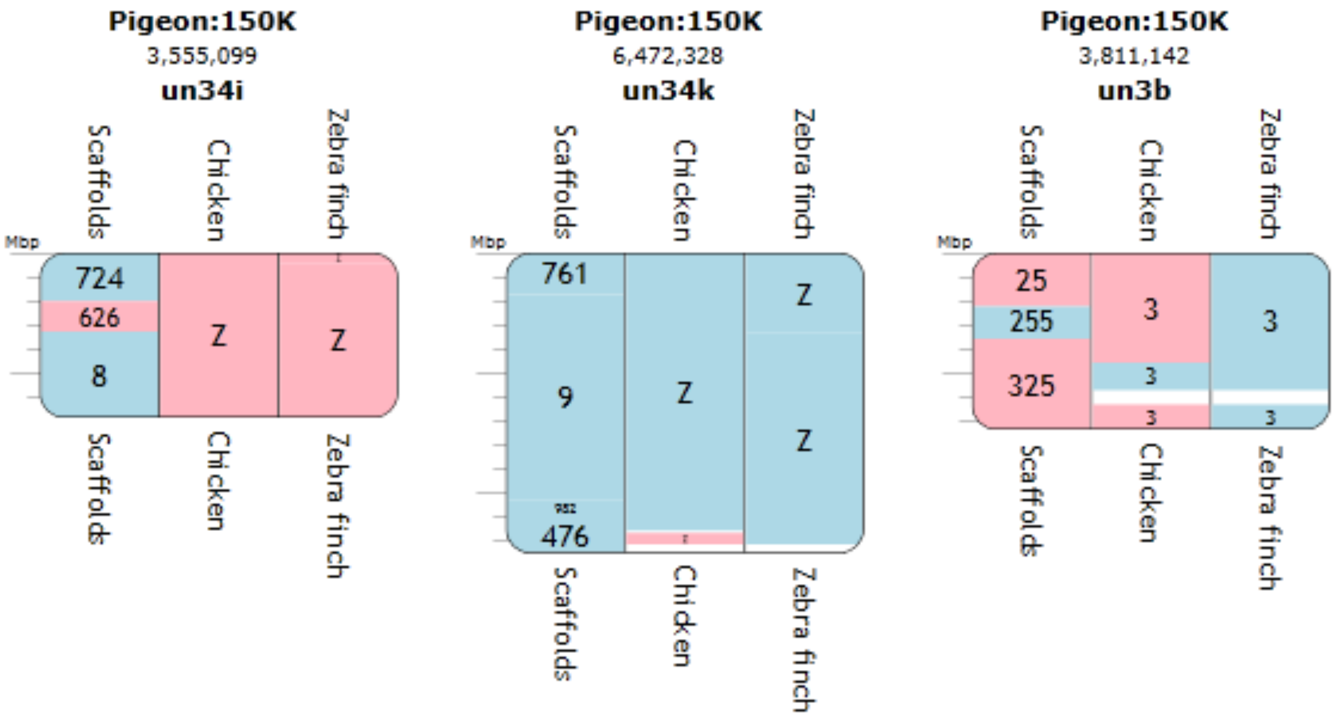

Pigeon:150K
3,555,099
un34i
Scaffolds
Chicken
Zebra finch
Mbp
724
626
8
Z
Z
Scaffolds
Chicken
Zebra finch
Pigeon:150K
6,472,328
un34k
Scaffolds
Chicken
Zebra finch
Mbp
761
9
476
Z
Z
Z
Scaffolds
Chicken
Zebra finch
Pigeon:150K
3,811,142
un3b
Scaffolds
Chicken
Zebra finch
Mbp
25
255
325
3
3
3
3
3
Scaffolds
Chicken
Zebra finch

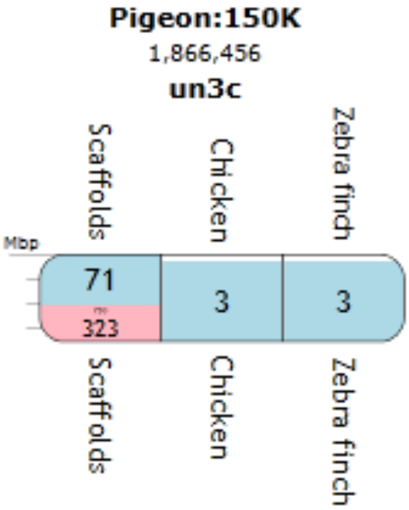

Pigeon:150K
1,866,456
un3c
Scaffolds
Chicken
Zebra finch
Mbp
71
323
3
3
Scaffolds
Chicken
Zebra finch

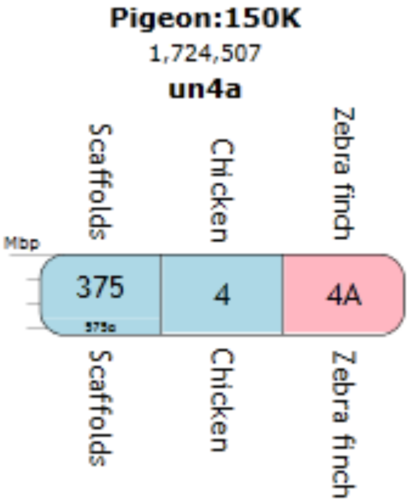

Pigeon:150K
1,724,507
un4a
Scaffolds
Chicken
Zebra finch
Mbp
375
4
4A
Scaffolds
Chicken
Zebra finch

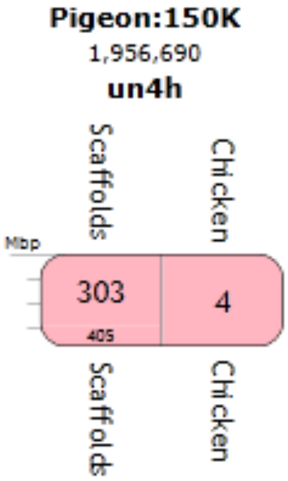

Pigeon:150K
1,956,690
un4h
Scaffolds
Chicken
Mbp
303
405
4
Scaffolds
Chicken

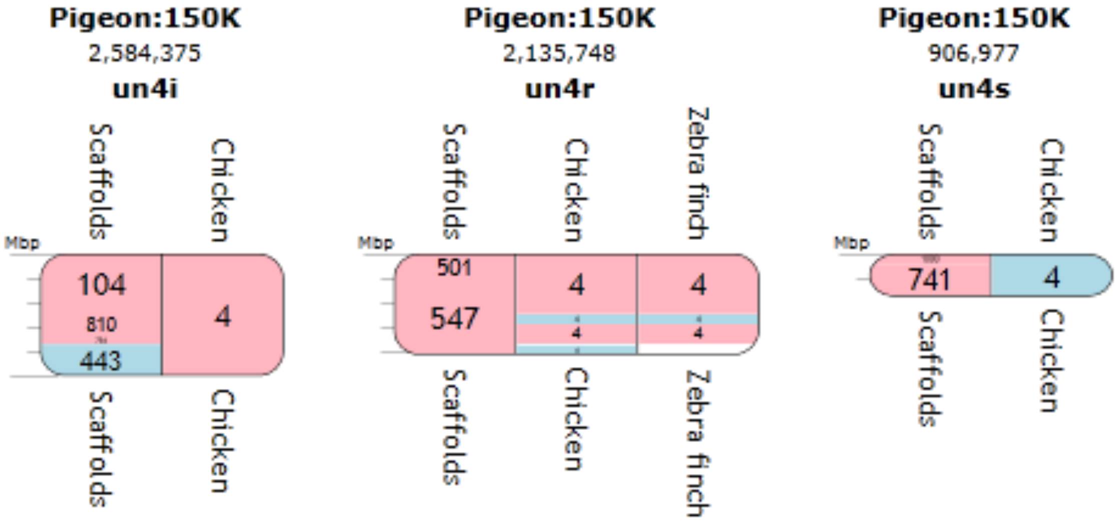

Pigeon:150K
2,584,375
un4i
Scaffolds
Chicken
Mbp
104
810
443
4
Scaffolds
Chicken
Pigeon:150K
2,135,748
un4r
Scaffolds
Chicken
Zebra finch
Mbp
501
547
4
4
4
4
Scaffolds
Chicken
Zebra finch
Pigeon:150K
906,977
un4s
Scaffolds
Chicken
Mbp
741
4
Scaffolds
Chicken

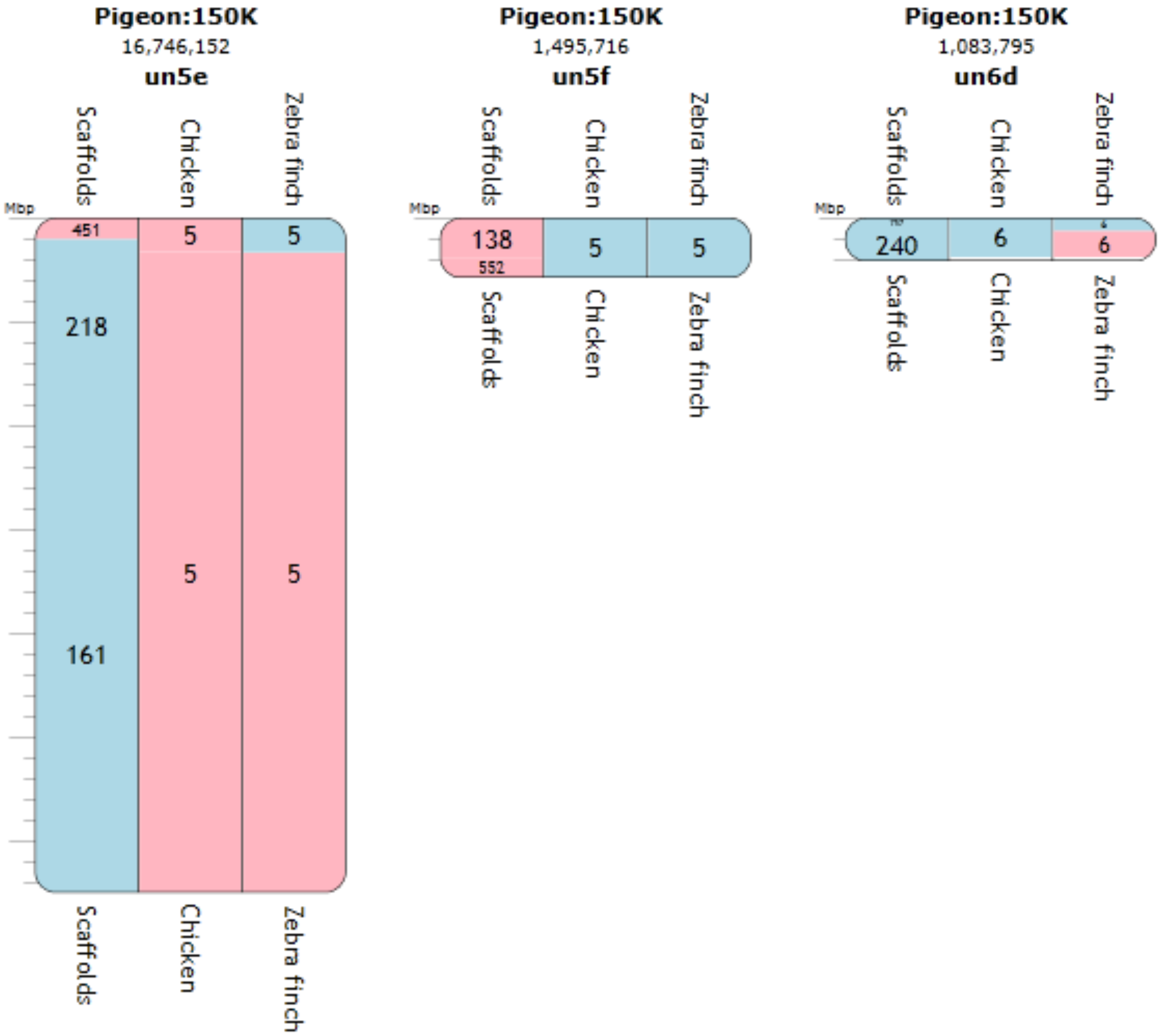
Pigeon:150K
16,746,152
un5e
Scaffolds
Chicken
Zebra finch
Mbp
451
5
5
218
5
5
161
Scaffolds
Chicken
Zebra finch
Pigeon:150K
1,495,716
un5f
Scaffolds
Chicken
Zebra finch
Mbp
138
552
5
5
Scaffolds
Chicken
Zebra finch
Pigeon:150K
1,083,795
un6d
Scaffolds
Chicken
Zebra finch
Mbp
240
6
6
Scaffolds
Chicken
Zebra finch

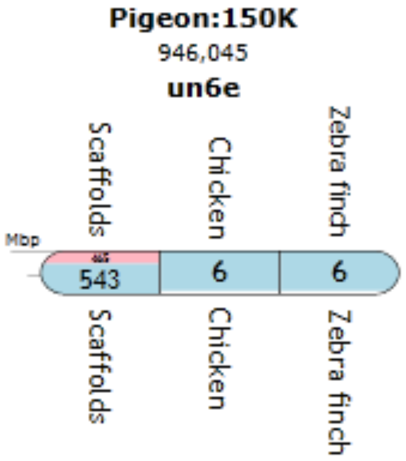
Pigeon:150K
946,045
un6e
Mbp
Scaffolds
Chicken
Zebra finch
543
6
6
Scaffolds
Chicken
Zebra finch

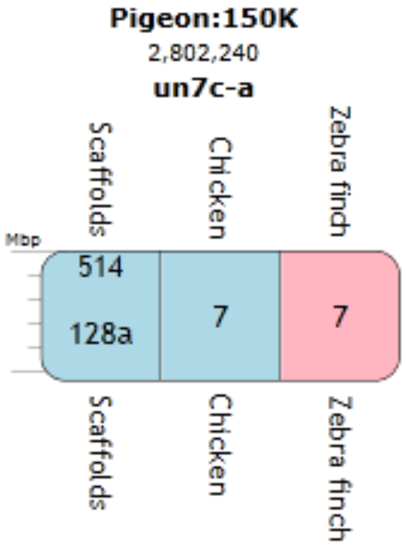
Pigeon:150K
2,802,240
un7c-a
Mbp
Scaffolds
Chicken
Zebra finch
514
128a
7
7
Scaffolds
Chicken
Zebra finch

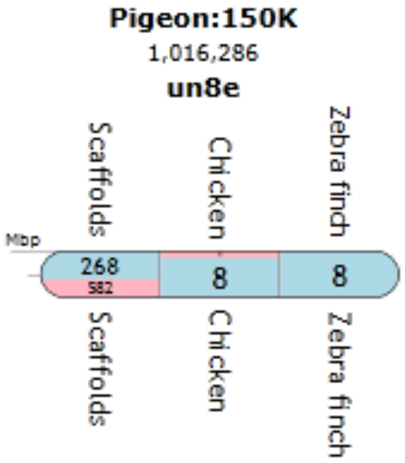
Pigeon:150K
1,016,286
un8e
Mbp
Scaffolds
Chicken
Zebra finch
268
582
8
8
Scaffolds
Chicken
Zebra finch

Pigeon:150K

3,819,946

un9b

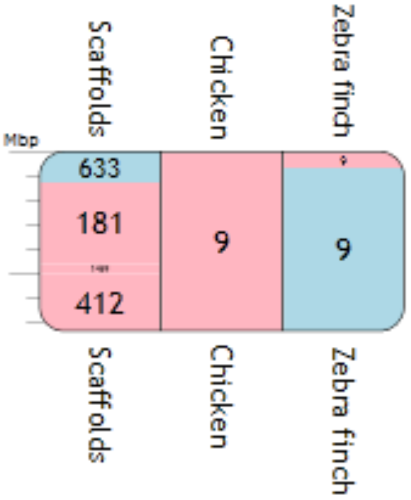

Supplemental Figure S4. Pigeon chromosomes and unplaced PCFs visualizations from Evolution Highway (http://eh-demo.ncsa.uiuc.edu/birds). Blue blocks represent “+” orientation, pink blocks denote “-“ orientation and grey blocks unknown (“?”) orientation. “BACs” track depicts the location of the BAC clones used to place and orient predicted chromosome fragments (PCFs) along the chromosomes. “PCFs” track depicts the position and orientation of the PCFs obtained from RACA (Kim et al. 2013). “Scaffolds” track depicts the position and orientation of the Cliv_1.0 scaffolds (Shapiro et al. 2013). “Chicken” and “Zebra finch” tracks depict the pairwise homologous synteny blocks (HSBs) between pigeon and chicken, pigeon and zebra finch genomes, respectively.
