# Supplementary material for: Upgrading short-read animal genome assemblies to chromosome level using comparative genomics and a universal probe set
Source: Genome Res. 2017 May;27(5):875–84. doi: 10.1101/gr.213660.116 (PMC5411781; doi:10.1101/gr.213660.116)
Supplement: Supplemental Material [file supp_gr.213660.116_Supplemental_Fig_S5.pdf]

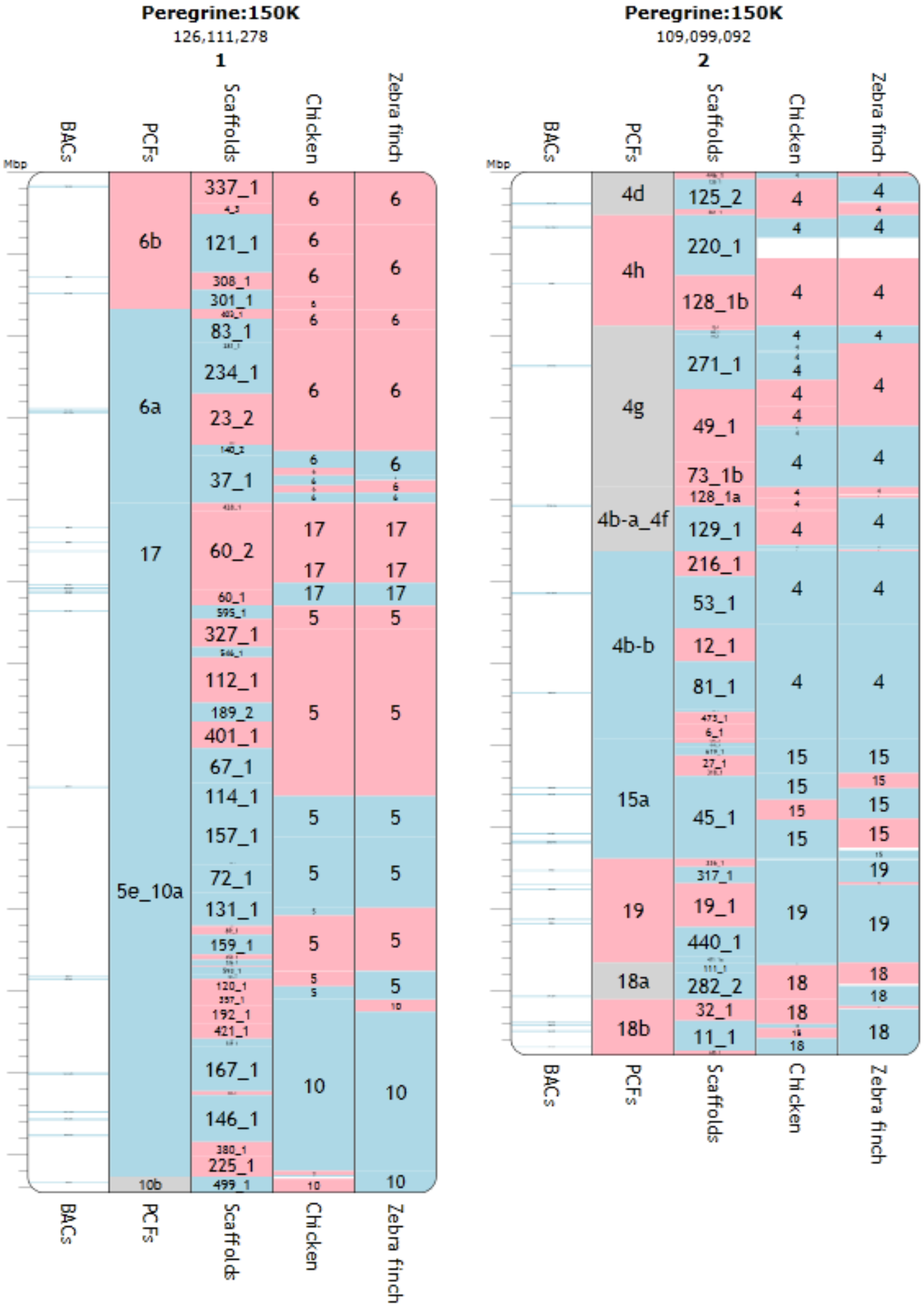

Peregrine:150K
126,111,278
1
BACs
PCFs
Scaffolds
Chicken
Zebra finch
Mbp
6b
6a
17
5e_10a
10b
337_1
121_1
308_1
301_1
83_1
234_1
23_2
37_1
60_2
60_1
595_1
327_1
112_1
189_2
401_1
67_1
114_1
157_1
72_1
131_1
159_1
120_1
192_1
421_1
167_1
146_1
380_1
225_1
499_1
6
17
5
10
Peregrine:150K
109,099,092
2
4d
4h
4g
4b-a_4f
4b-b
15a
19
18a
18b
125_2
220_1
128_1b
271_1
49_1
73_1b
128_1a
129_1
216_1
53_1
12_1
81_1
473_1
6_1
27_1
45_1
317_1
19_1
440_1
282_2
32_1
11_1
4
15
19
18

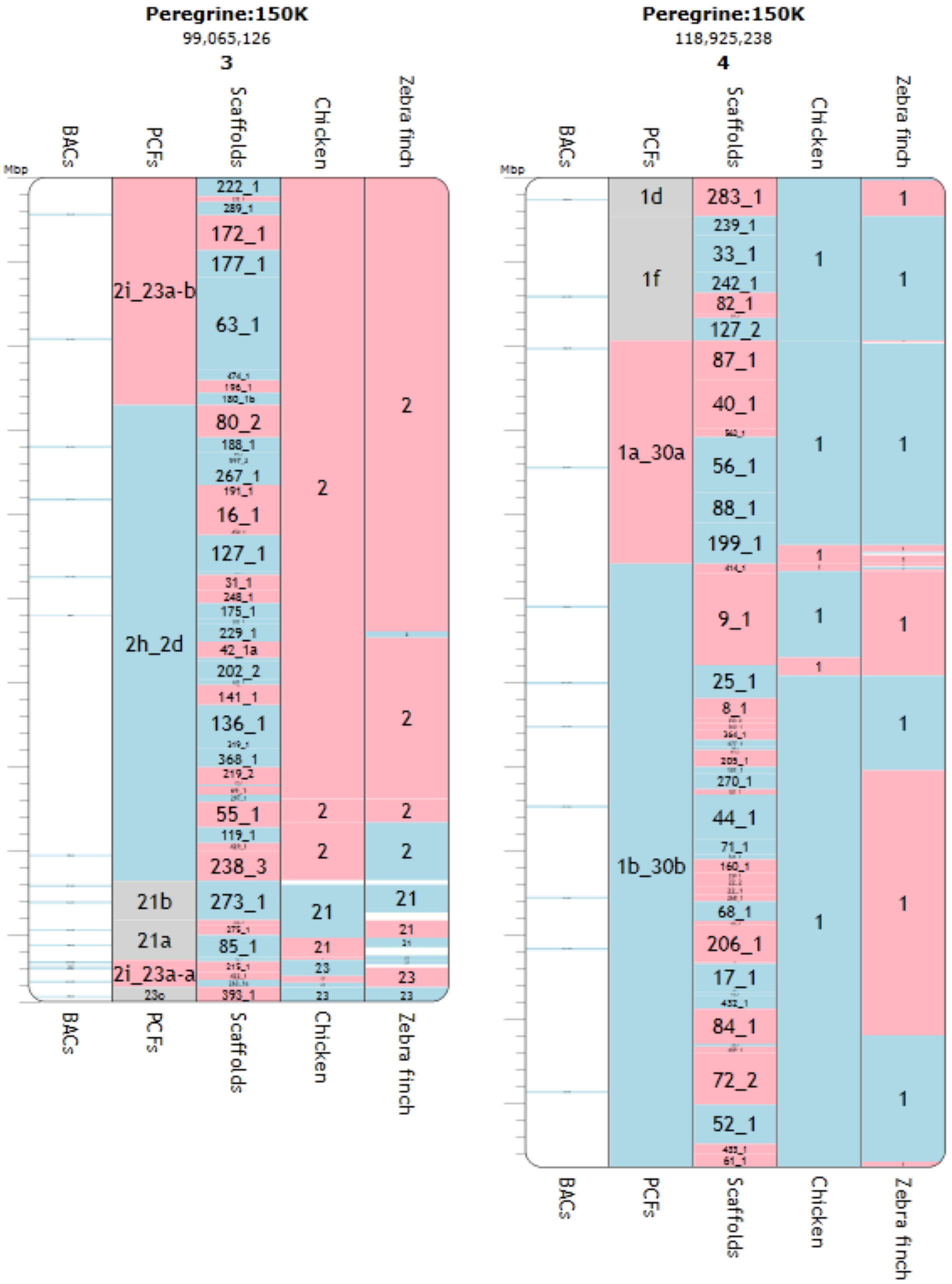

Peregrine:150K
99,065,126
3
BACs
PCFs
Scaffolds
Chicken
Zebra finch
Mbp
2i_23a-b
2h_2d
21b
21a
2i_23a-a
23c
222_1
289_1
172_1
177_1
63_1
196_1
80_2
188_1
267_1
191_1
16_1
127_1
31_1
248_1
175_1
229_1
42_1a
202_2
141_1
136_1
368_1
219_2
55_1
119_1
238_3
273_1
85_1
393_1
2
21
23
Peregrine:150K
118,925,238
4
1d
1f
1a_30a
1b_30b
283_1
239_1
33_1
242_1
82_1
127_2
87_1
40_1
56_1
88_1
199_1
9_1
25_1
8_1
270_1
44_1
71_1
160_1
68_1
206_1
17_1
452_1
84_1
72_2
52_1
61_1
1

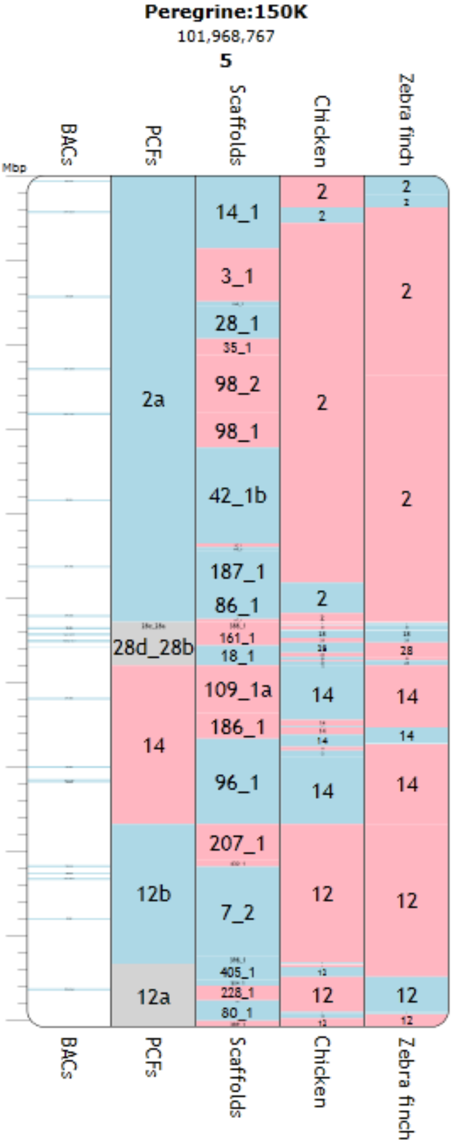
Peregrine:150K
101,968,767
5
Mbp
BACs
PCFs
Scaffolds
Chicken
Zebra finch
2a
28d_28b
14
12b
12a
14_1
3_1
28_1
35_1
98_2
98_1
42_1b
187_1
86_1
161_1
18_1
109_1a
186_1
96_1
207_1
7_2
405_1
228_1
80_1
2
14
12
28

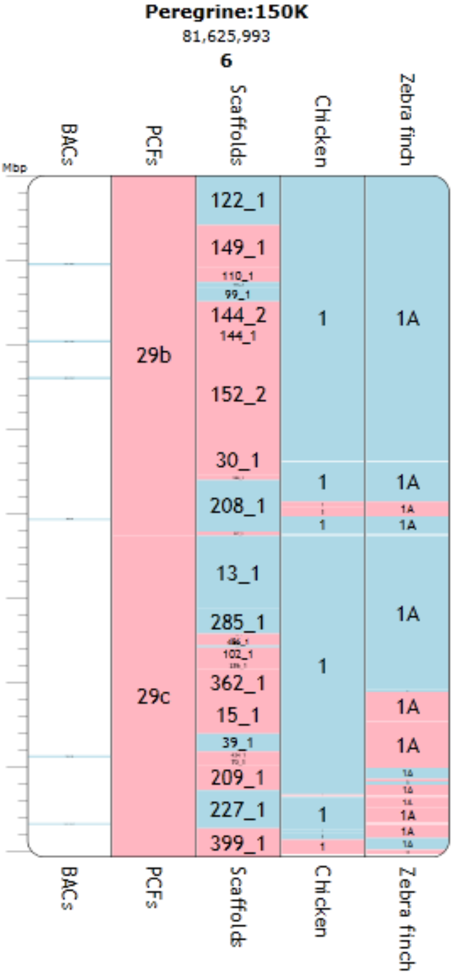
Peregrine:150K
81,625,993
6
Mbp
BACs
PCFs
Scaffolds
Chicken
Zebra finch
29b
29c
122_1
149_1
110_1
99_1
144_2
144_1
152_2
30_1
208_1
13_1
285_1
102_1
362_1
15_1
39_1
209_1
227_1
399_1
1
1A

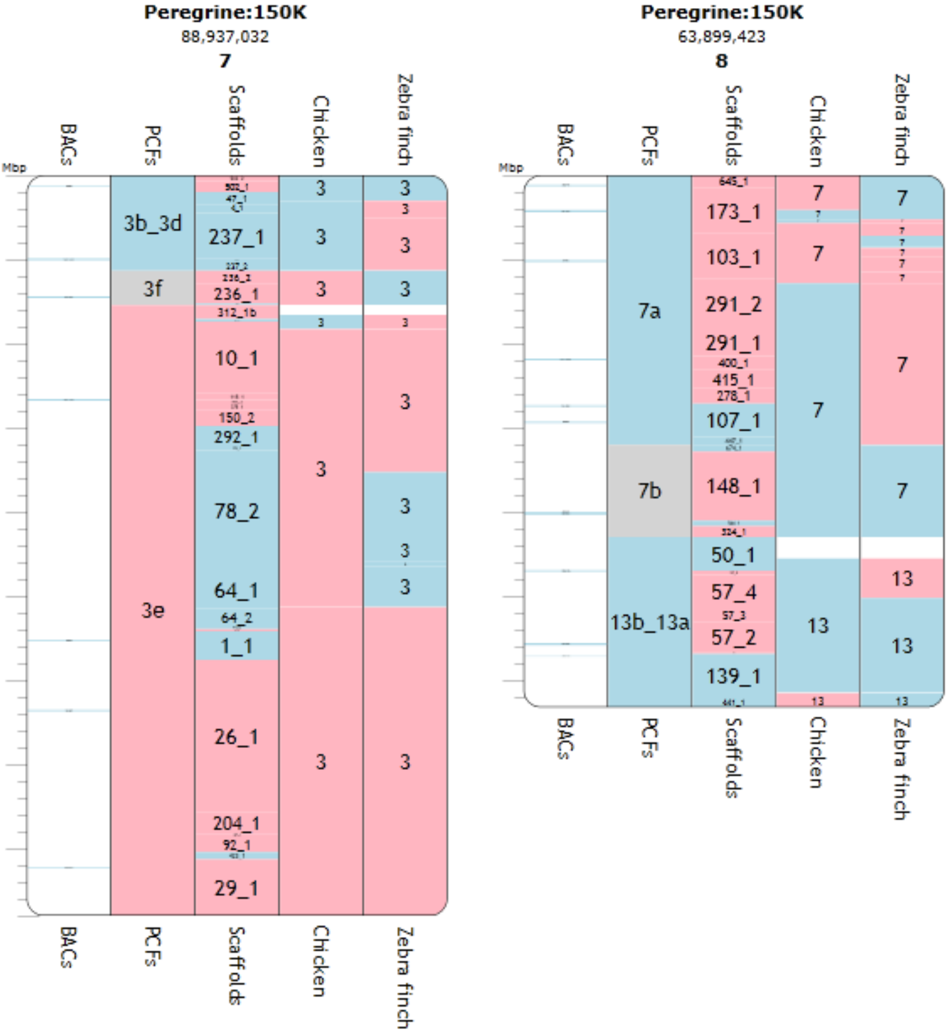

Peregrine:150K
88,937,032
7
Mbp
BACs
PCFs
Scaffolds
Chicken
Zebra finch
3b_3d
3f
3e
502_1
47_1
237_1
237_2
236_2
236_1
312_1b
10_1
150_2
292_1
78_2
64_1
64_2
1_1
26_1
204_1
92_1
29_1
3
Peregrine:150K
63,899,423
8
7a
7b
13b_13a
645_1
173_1
103_1
291_2
291_1
400_1
415_1
278_1
107_1
148_1
324_1
50_1
57_4
57_3
57_2
139_1
441_1
7
13

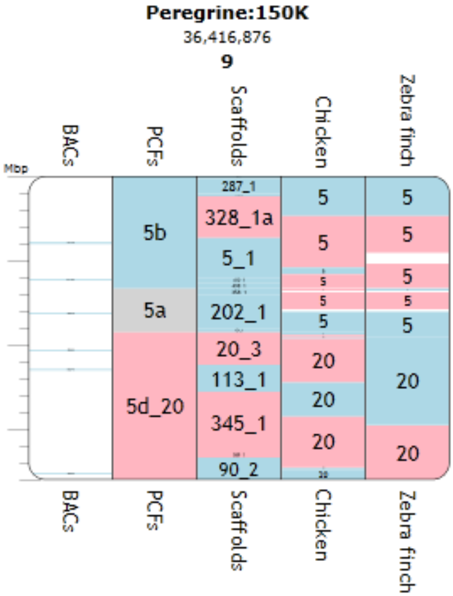

Peregrine:150K
36,416,876
9
BACs
PCFs
Scaffolds
Chicken
Zebra finch
Mbp
5b
5a
5d_20
287_1
328_1a
5_1
202_1
20_3
113_1
345_1
90_2
5
5
5
5
5
20
20
20
5
5
5
5
5
20
20

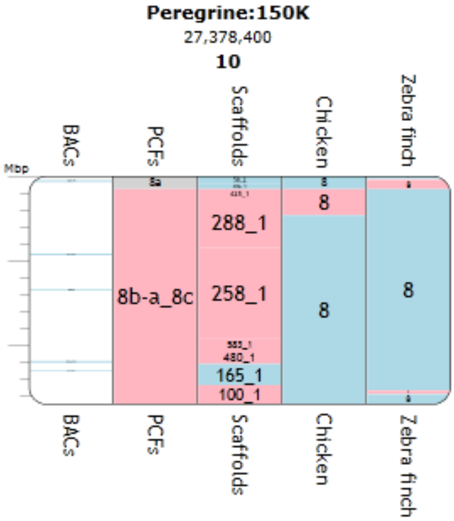

Peregrine:150K
27,378,400
10
BACs
PCFs
Scaffolds
Chicken
Zebra finch
Mbp
8a
8b-a_8c
288_1
258_1
480_1
165_1
100_1
8
8
8
8

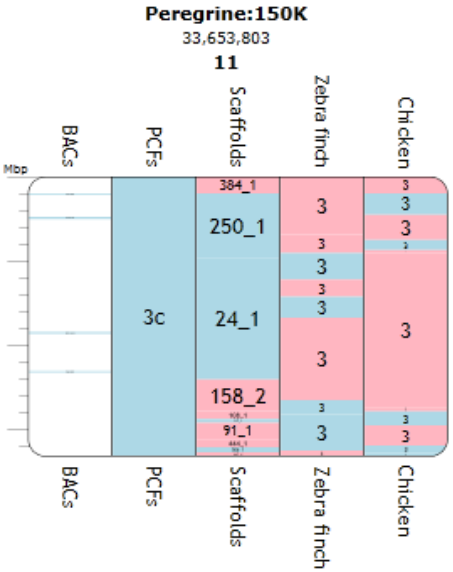

Peregrine:150K
33,653,803
11
BACs
PCFs
Scaffolds
Zebra finch
Chicken
Mbp
3c
384_1
250_1
24_1
158_2
91_1
3

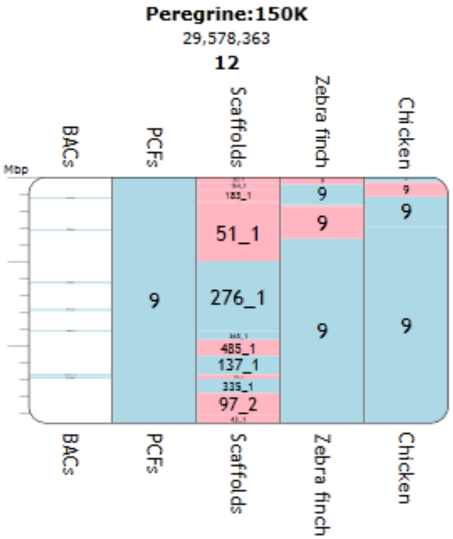

Peregrine:150K
29,578,363
12
BACs
PCFs
Scaffolds
Zebra finch
Chicken
Mbp
9
51_1
276_1
485_1
137_1
335_1
97_2

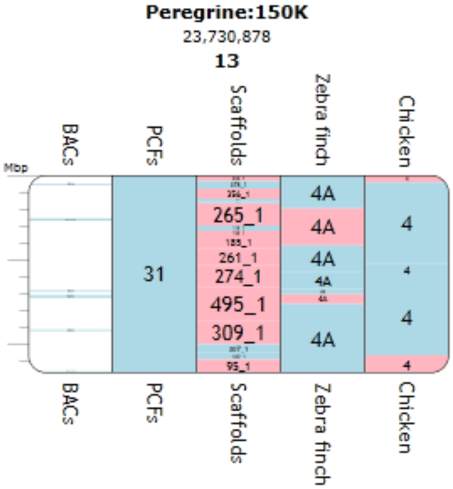
Peregrine:150K
23,730,878
13
BACs
PCFs
Scaffolds
Zebra finch
Chicken
Mbp
31
265_1
261_1
274_1
495_1
309_1
95_1
4A
4A
4A
4A
4A
4
4
4
4
BACs
PCFs
Scaffolds
Zebra finch
Chicken

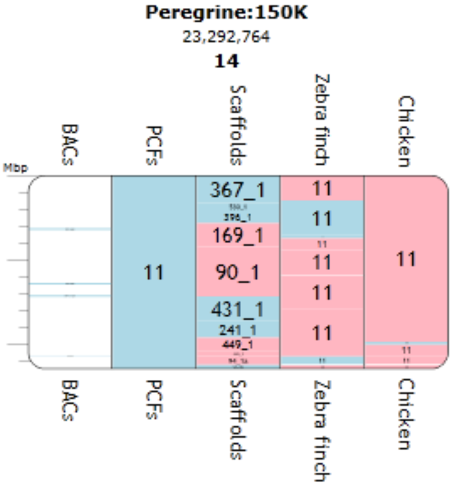
Peregrine:150K
23,292,764
14
BACs
PCFs
Scaffolds
Zebra finch
Chicken
Mbp
11
367_1
169_1
90_1
431_1
241_1
449_1
11
11
11
11
11
11
BACs
PCFs
Scaffolds
Zebra finch
Chicken

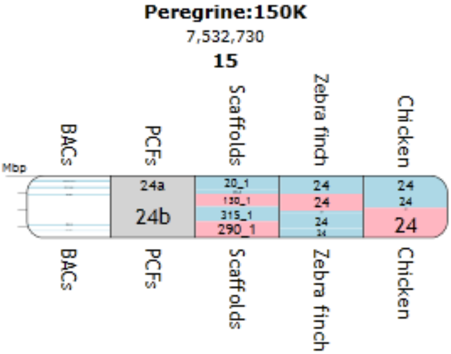
Peregrine:150K
7,532,730
15
BACs
PCFs
Scaffolds
Zebra finch
Chicken
Mbp
24a
24b
20_1
130_1
315_1
290_1
24
24
24
24
24
24
BACs
PCFs
Scaffolds
Zebra finch
Chicken

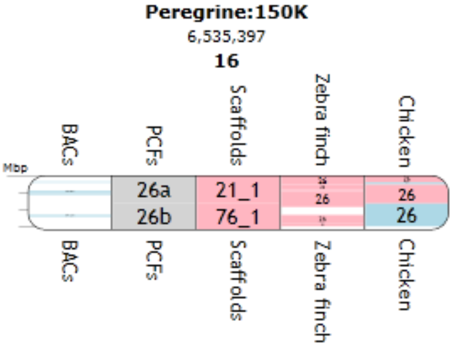
Peregrine:150K
6,535,397
16
BACs
PCFs
Scaffolds
Zebra finch
Chicken
Mbp
26a
26b
21_1
76_1
26
26
26
BACs
PCFs
Scaffolds
Zebra finch
Chicken

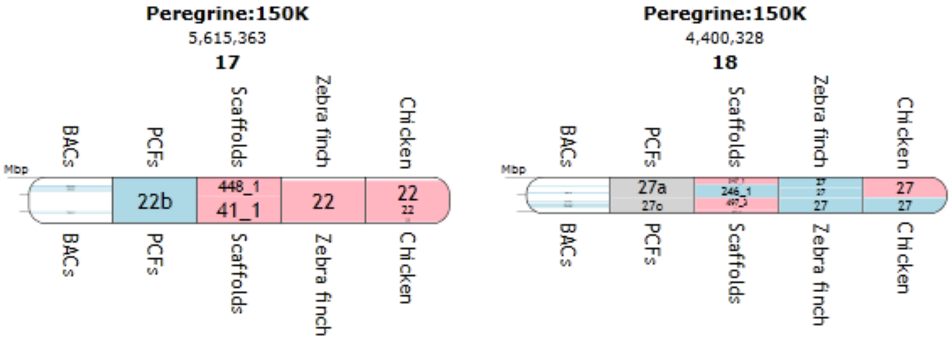
Peregrine:150K
5,615,363
17
BACs
PCFs
Scaffolds
Zebra finch
Chicken
Mbp
22b
448_1
41_1
22
22
22
Peregrine:150K
4,400,328
18
27a
27c
246_1
27
27
27

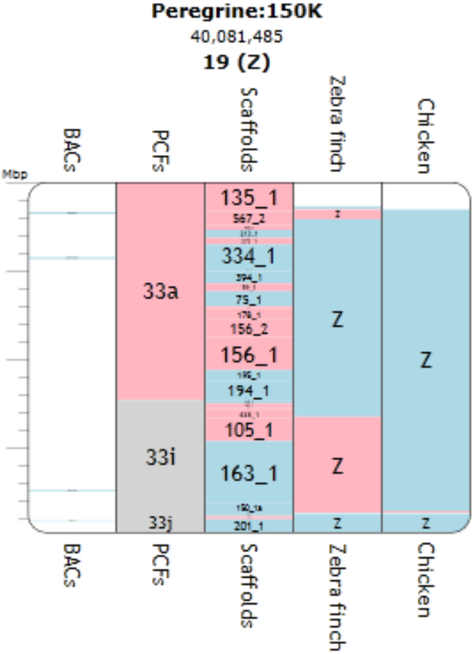
Peregrine:150K
40,081,485
19 (Z)
BACs
PCFs
Scaffolds
Zebra finch
Chicken
Mbp
33a
33i
33j
135_1
567_2
334_1
75_1
156_2
156_1
194_1
105_1
163_1
201_1
Z
Z
Z
Z
Z
Z
BACs
PCFs
Scaffolds
Zebra finch
Chicken

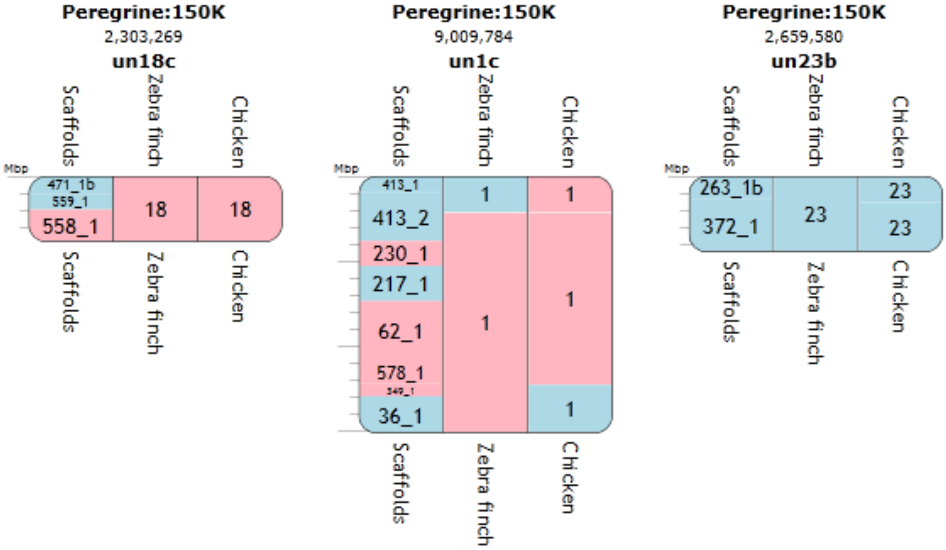
Peregrine:150K
2,303,269
un18c
Scaffolds
Zebra finch
Chicken
Mbp
471_1b
559_1
558_1
18
18
Peregrine:150K
9,009,784
un1c
Scaffolds
Zebra finch
Chicken
Mbp
413_1
413_2
230_1
217_1
62_1
578_1
36_1
1
1
1
1
1
Peregrine:150K
2,659,580
un23b
Scaffolds
Zebra finch
Chicken
Mbp
263_1b
372_1
23
23
23

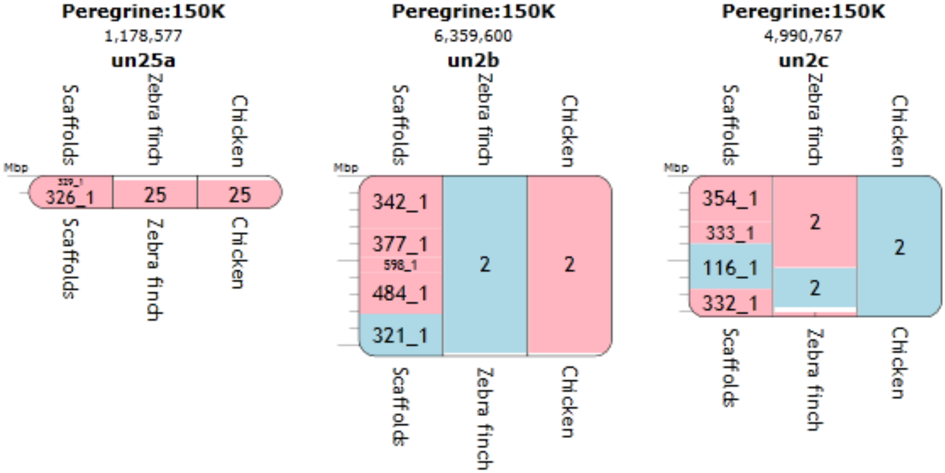
Peregrine:150K
1,178,577
un25a
Scaffolds
Zebra finch
Chicken
Mbp
326_1
25
25
Peregrine:150K
6,359,600
un2b
Scaffolds
Zebra finch
Chicken
Mbp
342_1
377_1
484_1
321_1
2
2
Peregrine:150K
4,990,767
un2c
Scaffolds
Zebra finch
Chicken
Mbp
354_1
333_1
116_1
332_1
2
2
2

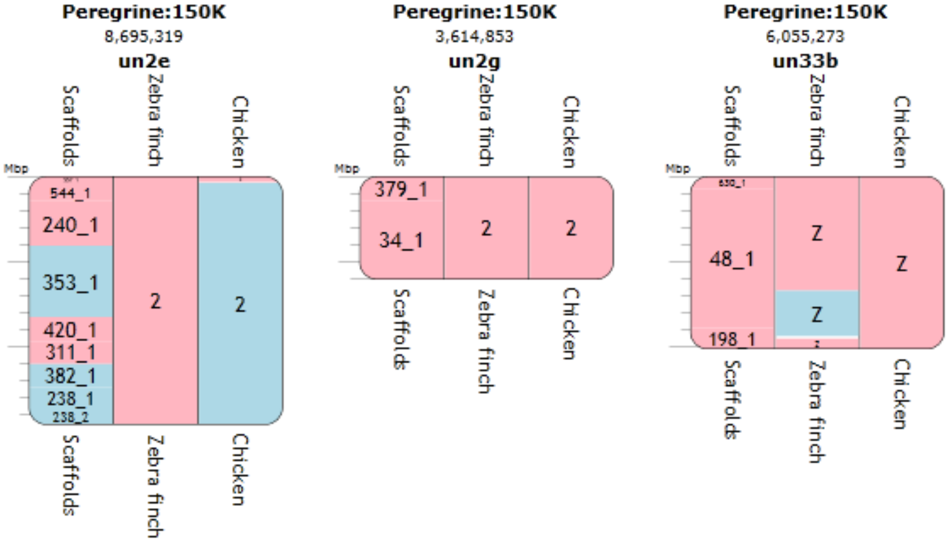
Peregrine:150K
8,695,319
un2e
Scaffolds
Zebra finch
Chicken
Mbp
544_1
240_1
353_1
420_1
311_1
382_1
238_1
238_2
2
2
Peregrine:150K
3,614,853
un2g
379_1
34_1
2
2
Peregrine:150K
6,055,273
un33b
48_1
198_1
Z
Z
Z

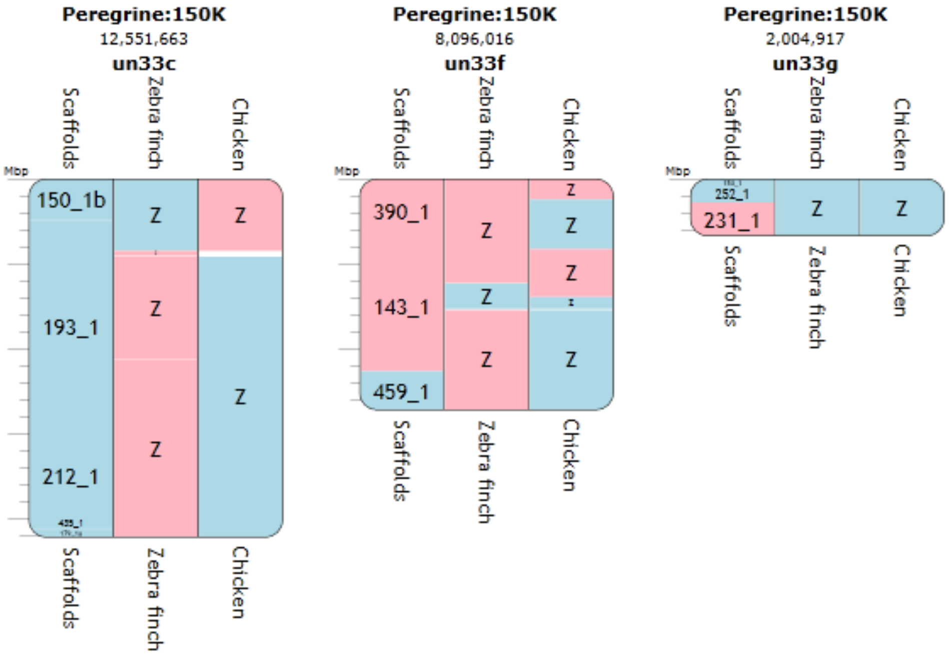

Peregrine:150K
12,551,663
un33c
Scaffolds
Zebra finch
Chicken
Mbp
150_1b
193_1
212_1
Z
Peregrine:150K
8,096,016
un33f
390_1
143_1
459_1
Peregrine:150K
2,004,917
un33g
252_1
231_1

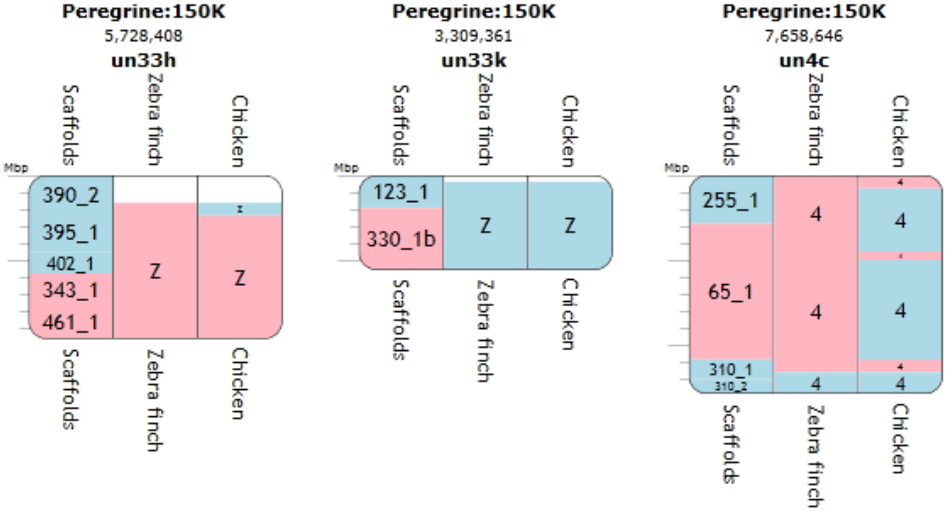

Peregrine:150K
5,728,408
un33h
Scaffolds
Zebra finch
Chicken
Mbp
390_2
395_1
402_1
343_1
461_1
Z
Z
Peregrine:150K
3,309,361
un33k
123_1
330_1b
Z
Z
Peregrine:150K
7,658,646
un4c
255_1
65_1
310_1
310_2
4

Supplemental Figure S5. Falcon chromosomes and unplaced PCFs visualizations from Evolution Highway (http://eh-demo.ncsa.uiuc.edu/birds). Blue blocks represent “+” orientation, pink blocks denote “-“ orientation and grey blocks unknown (“?”) orientation. “BACs” track depicts the location of the BAC clones used to place and orient predicted chromosome fragments (PCFs) along the chromosomes. “PCFs” track depicts the position and orientation of the PCFs obtained from RACA (Kim et al. 2013). “Scaffolds” track depicts the position and orientation of the F_peregrinus_v1.0 scaffolds (Zhan et al. 2013). “Chicken” and “Zebra finch” tracks depict the pairwise homologous synteny blocks (HSBs) between falcon and chicken, falcon and zebra finch genomes, respectively.
