# Supplementary material for: Upgrading short-read animal genome assemblies to chromosome level using comparative genomics and a universal probe set
Source: Genome Res. 2017 May;27(5):875–84. doi: 10.1101/gr.213660.116 (PMC5411781; doi:10.1101/gr.213660.116)
Supplement: Supplemental Material [file supp_gr.213660.116_Supplemental_Material.doc]

**Table of Contents**

[Supplemental Methods 2](#__RefHeading___Toc465674518)

[Pairwise and multiple genome alignments, nucleotide evolutionary conservation scores and conserved elements 2](#__RefHeading___Toc465674519)

[Reference-assisted chromosome assembly of pigeon and falcon genomes 3](#__RefHeading___Toc465674520)

[PCR amplification 4](#__RefHeading___Toc465674521)

[Supplemental Results 5](#__RefHeading___Toc465674522)

[Comparative visualization of pigeon and falcon chromosome assemblies 5](#__RefHeading___Toc465674523)

[Falcon chromosome assembly 5](#__RefHeading___Toc465674524)

[Supplemental Tables 7](#__RefHeading___Toc465674525)

[Supplemental Table S1. Number of scaffolds split by RACA when using chicken or zebra finch genomes as reference for pigeon PCF reconstructions. 7](#__RefHeading___Toc465674526)

[Supplemental Table S2. Statistics for the scaffold split regions tested by PCR. 8](#__RefHeading___Toc465674527)

[Supplemental Table S3. DNA sequence feature information returned from the BAC clone analysis pipeline. 9](#__RefHeading___Toc465674528)

[Supplemental Table S4. Avian BAC clones and expected FISH success rates for the phylogenetically distant species (divergence time ≥69 MY). 10](#__RefHeading___Toc465674529)

[Supplemental Table S8: Peregrine falcon and pigeon lineage-specific EBRs. 11](#__RefHeading___Toc465674530)

[Supplemental Table S9. Comparison and ratios of the number of bases from populated transposable elements in 10 kb intervals overlapping falcon EBRs and the rest of the falcon genome. 12](#__RefHeading___Toc465674531)

[Supplemental Table S11. Significant Mann-Whitney U comparisons of CNE densities for avian lineage-specific EBRs and their four adjacent intervals (**±**2) of the same size. 13](#__RefHeading___Toc465674532)

[Supplemental Table S12. Statistics for CNE density in 1 kb windows for avian EBRs, msHSBs, and genome-wide. 14](#__RefHeading___Toc465674533)

[Supplemental Table S13. Significant Mann-Whitney U comparisons for distances between zero and high CNE density windows. 15](#__RefHeading___Toc465674534)

[Supplemental Figures 16](#__RefHeading___Toc465674535)

[Supplemental Figure S1. Classification tree used to predict the non-successful (0) or successful hybridization (1) of a BAC clone on at least one phylogenetically distant species (divergence time ≥69 MY). 16](#__RefHeading___Toc465674536)

[Supplemental Figure S2. Number of successful zoo-FISH experiments for selected and random BAC clone sets with phylogenetically distant species metaphases (divergence time ≥69 MY). Number above each bar depict number of BAC clones in each category. 17](#__RefHeading___Toc465674537)

[Supplemental Figure S3. Histogram of the spacing between universal BAC clones along the chicken genome. 18](#__RefHeading___Toc465674538)

[Supplemental Figure S6. Distance between zero and high CNE density windows (of msHSB density or higher) by window type. 19](#__RefHeading___Toc465674539)

[Supplemental References 20](#__RefHeading___Toc465674540)

Supplemental Methods

Pairwise and multiple genome alignments, nucleotide evolutionary conservation scores and conserved elements

Pairwise alignments using chicken and zebra finch chromosome assemblies as references and all other assemblies as targets were generated with *LastZ* (v.1.02.00; Harris 2007) using the following parameters: *C=0 E=30 H=2000 K=3000 L=2200 O=400*. The resulting pairwise alignments were converted into the UCSC “chains” and “nets” alignment formats with axtChain (Kent et al. 2003; parameters: *-minScore=1000 -linearGap=medium -verbose=0*) followed by chainAntiRepeat, chainSort, chainPreNet, chainNet and netSyntenic, all with default parameters. Chicken chromosome as reference net alignments for 21 avian genomes were used to build multiple alignment files (MAF) with MULTIZ (Blanchette et al. 2004). The evolutionary conservation scores and DNA conserved elements (CEs) for all chicken nucleotides assigned to chromosomes were estimated using PhastCons (Hubisz et al. 2011) with the following parameters: *expected-length=45, target-coverage=0.3 and rho=0.2506*. Conserved non-coding elements obtained from the alignments of 48 avian genomes were used (Farré et al. 2016).

Reference-assisted chromosome assembly of pigeon and falcon genomes

Pigeon and falcon predicted chromosome fragments (PCFs) were generated using the Reference-Assisted Chromosome Assembly (RACA; Kim et al. 2013) tool. The target genome mate pair and paired read mappings to the corresponding scaffolds required for RACA were done with Bowtie2 (v2.0.1; Langmead and Salzberg 2012) using the parameters suitable for each individual sequencing library. For the libraries with read length ≤90 bp the following parameters were used: *-N 1 -3 5 --no-discordant* and for reads >90 bp: *-N 1 -3 30 --no-discordant.* In addition, for mate-pair libraries (>2 kb insert size) we used *--rf*. Read-pairs whose mapping distance was more than (library insert size) ± (0.5 x insert size) were discarded. We chose zebra finch genome as reference and chicken as outgroup for falcon based on the phylogenetic distances between the species (Jarvis et al. 2014). For pigeon both chicken as reference and zebra finch as outgroup and vice versa experiments were performed because pigeon is phylogenetically distant from chicken and zebra finch. Two rounds of RACA were done for both species. The initial run was performed using the following parameters: *WINDOWSIZE=10 RESOLUTION=150000 MIN_INTRACOV_PERC=5*. Prior to the second run of RACA we tested the scaffolds split during the initial RACA run using PCR amplification across the split intervals. Based on the PCR results we established thresholds for the minimum physical coverage across the syntenic fragment (SF) join intervals that allowed us to separate scaffolds that were likely to be chimeric from those that were likely to be real (Supplemental Table S2). These thresholds were used to update the MIN_INTRACOV_PERC parameter to ~3 in pigeon and ~27 in falcon on the second run of RACA. In addition, scaffolds with structures confirmed by PCR and agreeing with cytogenetic maps (even with the minimum physical coverage between SFs below threshold) were maintained intact during the final RACA run by their inclusion in RACA ‘reliable adjacencies’ file.

PCR amplification

PCR amplification was performed in a volume of 10μL as follows: 5μL of Taq PCR Master Mix (Qiagen; for expected PCR product lengths up to 2 kb) or DreamTaq Master Mix (Fermentas; for expected product lengths from 2 to 6 kb), 1μL of each primer at 2μM and ≈30ng DNA. PCR amplification was carried out in a T100 Thermal Cycler (BioRad) using the following profile: initial denaturation at 95°C for 3 min, 35 cycles for 30 sec at 94°C, 1 min/kb at 55-60°C and 1 min at 72°C, and final extension at 72°C for 10 min. DNA fragments were stained with SYBR Safe (Invitrogen), separated in a 1.5% (for expected PCR product lengths up to 2 kb) or 1% (for expected PCR product lengths from 2 to 6 kb) agarose gel and visualized in a ChemiDOC MP system (Biorad).

Supplemental Results

Comparative visualization of pigeon and falcon chromosome assemblies

Comparative visualizations of both newly assembled genomes are available from the Evolution Highway comparative chromosome browser under the reference names “Peregrine:150K” and “Pigeon:150K” (http://eh-demo.ncsa.uiuc.edu/birds). We named falcon chromosomes 1-13 and Z according to (Nishida et al. 2008), and chromosomes 14-18 were numbered by decreasing combined length of the placed PCFs. Pigeon chromosomes 1-9 and Z were named according to (Hansmann et al. 2009) with the remaining chromosome names assigned according to chicken homeologues. Unassigned PCFs were named after their reference chromosome homeologues with “un” added in front of their names to distinguish from chromosome assemblies.

Falcon chromosome assembly

Each of the chicken largest macrochromosome homeologues (GGA1 to GGA5) were split across two falcon chromosomes (Fig. 3B). The falcon GGA1 and GGA3 counterparts were represented as two entire chromosomes each (FPE4 and FPE6, FPE7 and FPE11, respectively). GGA2 was split across FPE3 and FPE5, both of which exhibited additional fusions of microchromosomes with GGA21 and 23 fused in FPE3 and GGA12, 14 and 28 fused in FPE5. Consistent with the pigeon assembly results (and the majority of birds), GGA4 was found to be split across two falcon chromosomes (FPE2 and FPE13), the former of which exhibited three additional microchromosomal fusions (GGA15, 18 and 19). Both GGA6 and GGA7 homeologues were found as single blocks fused with other chicken chromosome material within falcon chromosomes FPE1 and FPE8 respectively. Among the other chicken macrochromosomes, only GGA8 and GGA9 were represented as individual chromosomes (FPE10 and FPE12, respectively). Of the 17 mapped chicken microchromosomes, 11 were fused with other chromosomes. A total of 69 intrachromosomal rearrangements were detected in the falcon lineage (Supplemental Table S8).

Supplemental Tables

Supplemental Table S1. Number of scaffolds split by RACA when using chicken or zebra finch genomes as reference for pigeon PCF reconstructions.

|  | **Chicken** | **Zebra finch** |
| --- | --- | --- |
| No. scaffolds used | 572 (100%) | 564 (100%) |
| No. split scaffolds | 17 (2.97%) | 22 (3.90%) |
| No. scaffolds unique for the reference | 1 (5.88%) | 8 (36.36%) |

Supplemental Table S2. Statistics for the scaffold split regions tested by PCR.

| **Statistics** | **Peregrine** | **Pigeon** |
| --- | --- | --- |
| Pair-end read physical coverage within scaffolds | 9-983 | 3-416 |
| No. split SF adjacencies by RACA (default param.) | 85 | 109 |
| No. tested scaffold split regions | 49 (100%) | 69 (100%) |
| No. amplified split regions (confirmed SF joints) | 41 (83.67%) | 58 (84.06%) |
| No. non-amplified split regions | 8 (16.33%) | 11 (15.94%) |
| No. tested RACA-suggested adjacencies | 4 | 7 |
| No. amplified adjacencies (chimeric SF joints) | 2 | 7 |
| No. non-amplified adjacencies | 2 | 0 |
| Final no. ambiguous SF joints from tested split regions | 6 | 4 |
| Selected pair-end read spanning threshold | 583 | 85 |
| No. tested split regions found below selected threshold | 16 (100%) | 7 (100%) |
| No. chimeric SF joints | 2 (12.50%) | 3 (42.86%) |
| No. confirmed SF joints | 9 (56.25%) | 3 (42.86%) |
| No. ambiguous SF joints | 5 (31.25%) | 1 (14.29%) |
| No. tested split regions found above selected threshold | 33 (100%) | 62 (100%) |
| No. chimeric SF joints | 0 (0%) | 4 (6.45%) |
| No. confirmed SF joints | 32 (96.97%) | 55 (88.71%) |
| No. ambiguous SF joints | 1 (3.03%) | 3 (4.84%) |

Supplemental Table S3. DNA sequence feature information returned from the BAC clone analysis pipeline.

| **Group** | **Feature** |
| --- | --- |
| General | BAC clone length |
|  | Percentage of a BAC clone missed nucleotides (Ns) |
| DNA conservation | Percentage of a BAC clone sequence with conservation scores |
|  | Average conservation score (only positions with score considered) |
|  | Average conservation score (positions without score included as 0) |
|  | Percentage of a BAC clone within conserved elements |
|  | Minimum length of conserved elements |
|  | Maximum length of conserved elements |
|  | Average length of conserved elements |
|  | Median of the length of conserved elements |
|  | Percentage of a BAC clone within conserved elements of length ≥ 100nt |
|  | Percentage of a BAC clone within conserved elements of length ≥ 200nt |
|  | Percentage of a BAC clone within conserved elements of length ≥ 300nt |
|  | Percentage of a BAC clone within conserved elements of length ≥ 400nt |
|  | Percentage of a BAC clone within conserved elements of length ≥ 500nt |
| Gene  content | Percentage of a BAC clone containing chicken genes |
|  | Percentage of a BAC clone containing chicken exons |
|  | Minimum length of chicken exons |
|  | Maximum length of chicken exons |
|  | Average length of chicken exons |
|  | Median of the length of chicken exons |
|  | Percentage of a BAC clone containing chicken exons of length ≥ 100nt |
|  | Percentage of a BAC clone containing chicken exons of length ≥ 200nt |
|  | Percentage of a BAC clone containing chicken exons of length ≥ 300nt |
|  | Percentage of a BAC clone containing chicken exons of length ≥ 400nt |
|  | Percentage of a BAC clone containing chicken exons of length ≥ 500nt |
|  | Percentage of a BAC clone containing chicken-human ortholog genes |
| Repeat content | Percentage of a BAC clone containing repetitive elements |
|  | Minimum length of repetitive elements |
|  | Maximum length of repetitive elements |
|  | Average length of repetitive elements |
|  | Median of the length of repetitive elements |
| GC  content | GC percentage of a BAC clone |
|  | GC percentage of highly conserved bases (conservation score ≥ 0.5) |
|  | Average GC percentage of conserved elements |

Supplemental Table S4. Avian BAC clones and expected FISH success rates for the phylogenetically distant species (divergence time ≥69 MY).

| **Library** | **Species** | **No. of analysed**  **BAC clones *** | **Obey CART**  **criteria** | **Expected**  **success rate** |
| --- | --- | --- | --- | --- |
| CHORI-260 | Turkey | 3,694 | 2,821 | 76.36 |
| CHORI-261 | Chicken | 40,410 | 30,399 | 75.23 |
| TGMCBA | Zebra finch | 80,009 | 61,335 | 76.66 |
| **Total/Average** |  | **124,113** | **94,555** | **76.18** |

* No. BAC clones after filtering steps. Clones mapped to chicken unplaced regions or linkage groups, clones smaller than 50 kb or longer than 300 kb were not included in the analysis.

Supplemental Table S8: Peregrine falcon and pigeon lineage-specific EBRs.

|  | **Peregrine falcon** | **Pigeon** |
| --- | --- | --- |
| Fusions | 13 | 0 |
| Fissions | 6 | 0 |
| Intrachromosomal | 69 | 70 |

Supplemental Table S9. Comparison and ratios of the number of bases from populated transposable elements in 10 kb intervals overlapping falcon EBRs and the rest of the falcon genome.

| Repeat set | **All EBRs** | | |  | **Intrachromosomal** | | |  | **Fusions** | | |  | **Fissions** | | |
| --- | --- | --- | --- | --- | --- | --- | --- | --- | --- | --- | --- | --- | --- | --- | --- |
|  | **EBR** | **non-EBR** | **Ratio** |  | **EBR** | **non-EBR** | **Ratio** |  | **EBR** | **non-EBR** | **Ratio** |  | **EBR** | **non-EBR** | **Ratio** |
| LINE | 212.11 | 272.40 | 0.78 |  | 224.80 | 272.37 | 0.82 |  | 147.00 | 272.35 | 0.54 |  | 213.83 | 272.32 | 0.78 |
| LINE-CR1 | 203.69 | 266.49 | 0.76 |  | 214.29 | 266.46 | 0.80 |  | 147.00 | 266.43 | 0.55 |  | 6.00 | 266.40 | 0.02 |
| LTR | 275.44 | 105.99 | 2.60 ***** |  | 307.23 | 106.01 | 2.90 ***** |  | 161.50 | 106.21 | 1.52 |  | 99.83 | 106.22 | 0.94 |
| LTR-ERV1 | 107.15 | 7.22 | 14.83 ***** |  | 118.58 | 7.24 | 16.38 ***** |  | 78.18 | 7.34 | 10.64 |  | 0.00 | 7.36 | 0.00 |

*****Statistically significant differences FDR corrected p-values <0.05. Only statistical significance for transposable elements covering ≥100 bp on average in the EBR and/or non-EBR 10 kb intervals in each individual comparison is reported.

Supplemental Table S11. Significant Mann-Whitney U comparisons of CNE densities for avian lineage-specific EBRs and their four adjacent intervals (±2) of the same size.

| **Group 1** | **Group 2** | **Median**  **Group 1** | **Median**  **Group 2** | **Ratio** | **p-value** |
| --- | --- | --- | --- | --- | --- |
| (± 2) | EBR | 0.021 | 0.001 | 21.778 | 1.56e-09 |
| Intra (± 2) | Intra EBR | 0.022 | 0.002 | 11.204 | 1.02e-08 |
| (+ 2) | EBR | 0.021 | 0.001 | 21.978 | 3.61e-08 |
| Intra (+ 2) | Intra EBR | 0.023 | 0.002 | 11.681 | 1.64e-07 |
| (± 1) | EBR | 0.015 | 0.001 | 15.948 | 3.35e-07 |
| (- 2) | EBR | 0.019 | 0.001 | 20.426 | 5.58e-07 |
| Intra (- 2) | Intra EBR | 0.022 | 0.002 | 10.924 | 2.15e-06 |
| Intra (± 1) | Intra EBR | 0.017 | 0.002 | 8.812 | 3.06e-06 |
| (+ 1) | EBR | 0.015 | 0.001 | 15.948 | 5.92e-06 |
| (- 1) | EBR | 0.016 | 0.001 | 16.378 | 1.32e-05 |
| Intra* | Inter^1^* | 0.016 | 0.001 | 12.240 | 2.40e-05 |
| Intra (+ 1) | Intra EBR | 0.017 | 0.002 | 8.771 | 3.46e-05 |
| Intra (- 1) | Intra EBR | 0.018 | 0.002 | 8.933 | 6.71e-05 |
| Intra* | Fusions* | 0.016 | 0.001 | 13.662 | 0.0002 |
| Intra (± 2) | Inter^1^ (± 2) | 0.022 | 0.003 | 8.475 | 0.0016 |
| Intra (± 2) | Fusions (± 2) | 0.022 | 0.002 | 11.911 | 0.0038 |
| Intra (+ 2) | Inter (+ 2) | 0.023 | 0.002 | 12.419 | 0.0233 |
| Intra (+ 2) | Fusions (+ 2) | 0.023 | 0.001 | 16.429 | 0.0270 |
| Intra (- 2) | Inter^1^ (- 2) | 0.022 | 0.003 | 6.255 | 0.0299 |
| Intra* | Fissions* | 0.016 | 0.003 | 5.350 | 0.0400 |
| Inter EBR | Intra EBR | 0.000 | 0.002 | 0.000 | 0.0445 |
| Intra (± 1) | Inter (± 1) | 0.017 | 0.003 | 6.081 | 0.0460 |

^1^ Fusions and fissions combined

* EBR and adjacent intervals were combined

Supplemental Table S12. Statistics for CNE density in 1 kb windows for avian EBRs, msHSBs, and genome-wide.

|  | **Average no. CNEs** | **Average no. CNE bases** | **Average density of CNE bases** | **Fraction ‘zero CNE windows’ (%)** |
| --- | --- | --- | --- | --- |
| Genome | 6.18 | 86.85 | 0.09 | 100 |
| msHSB | 7.54 | 106.81 | 0.11 | 15.44 |
| Intra | 1.90 | 23.72 | 0.02 | 0.41 |
| Fusion | 0.65 | 6.75 | 0.01 | 0.06 |
| Fission | 0.00 | 0.00 | 0.00 | 0.02 |
| EBR* | 1.71 | 21.25 | 0.02 | 0.50 |

*Fission, fusion and intrachromosomal EBRs combined.

Supplemental Table S13. Significant Mann-Whitney U comparisons for distances between zero and high CNE density windows.

| **Group 1** | **Group 2** | **Median**  **Group 1** | **Median**  **Group 2** | **Ratio** | **p-value** |
| --- | --- | --- | --- | --- | --- |
| Fission | Genome | 35 | 6 | 5.83 | <2.20e-16 |
| Fission | msHSB | 35 | 4 | 8.75 | <2.20e-16 |
| Fission | msHSB_long | 35 | 4 | 8.75 | <2.20e-16 |
| Fusion | Genome | 23 | 6 | 3.83 | <2.20e-16 |
| Fusion | msHSB | 23 | 4 | 5.75 | <2.20e-16 |
| Fusion | msHSB_long | 23 | 4 | 5.75 | <2.20e-16 |
| Intra | Genome | 19 | 6 | 3.17 | <2.20e-16 |
| Intra | msHSB | 19 | 4 | 4.75 | <2.20e-16 |
| Intra | msHSB_long | 19 | 4 | 4.75 | <2.20e-16 |
| msHSB | Genome | 4 | 6 | 0.67 | <2.20e-16 |
| msHSB_long | Genome | 4 | 6 | 0.67 | <2.20e-16 |
| Fission | Intra | 35 | 19 | 1.84 | 4.70e-14 |
| Fusion | Intra | 23 | 19 | 1.21 | 6.72e-05 |
| Fission | Fusion | 35 | 23 | 1.52 | 0.0038 |

Supplemental Figures

Supplemental Figure S1. Classification tree used to predict the non-successful (0) or successful hybridization (1) of a BAC clone on at least one phylogenetically distant species (divergence time ≥69 MY). For each tree node (A) represents the classification with higher representation on the data, and (B) proportions of BAC clones classified as 0 (left) and 1 (right) on the data. At each intermediate node, a case goes to the right child node if the condition is satisfied.

**
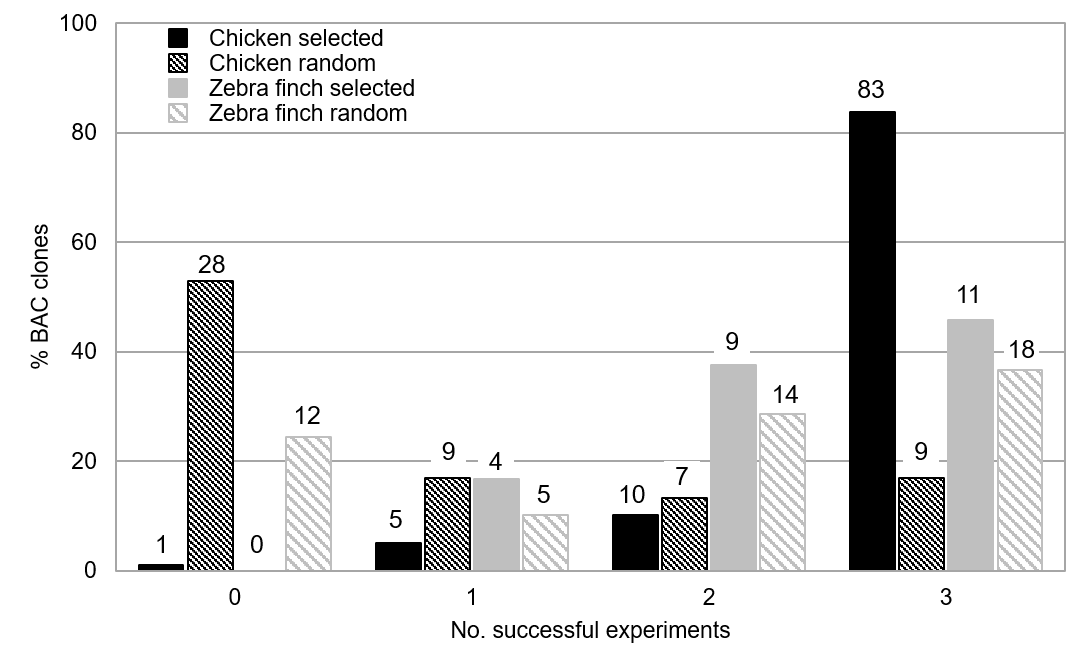
**

Supplemental Figure S2. Number of successful zoo-FISH experiments for selected and random BAC clone sets with phylogenetically distant species metaphases (divergence time ≥69 MY). Number above each bar depict number of BAC clones in each category.

164

44

13

6

2

2

2

Supplemental Figure S3. Histogram of the spacing between universal BAC clones along the chicken genome.


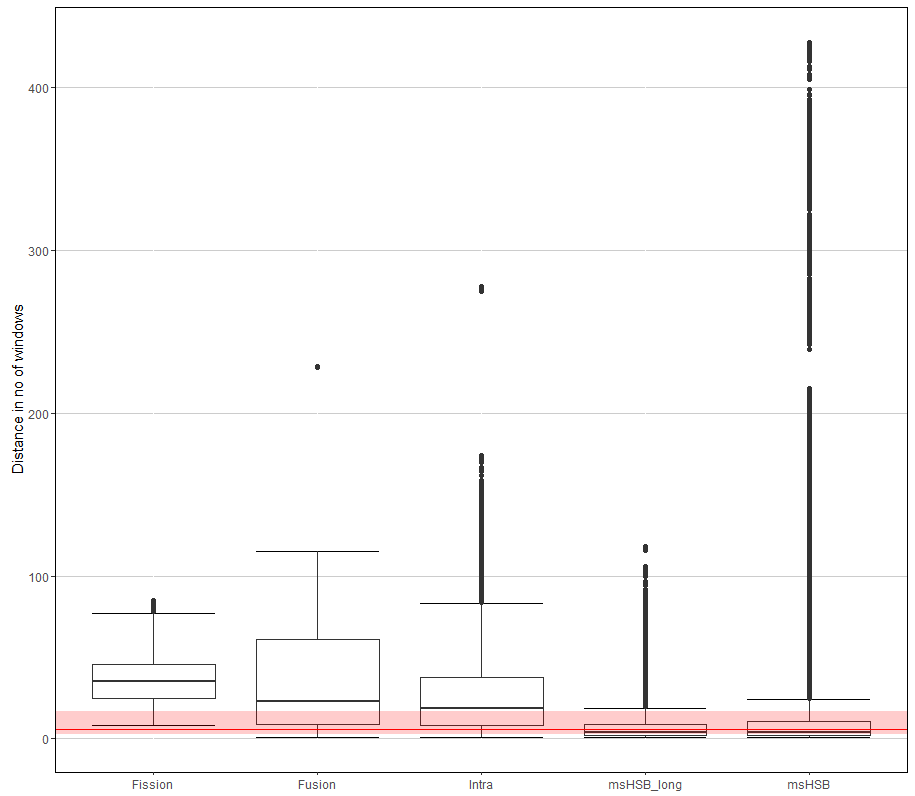


Supplemental Figure S6. Distance between zero and high CNE density windows (of msHSB density or higher) by window type. Red line represents whole genome median and red shade whole genome interquartile distance. ‘msHSB_long’ corresponds to the subset of msHSBs longer than expected under the random breakage of chromosomes in avian genomes and ‘msHSB’ to msHSB longer than 1.5 Mb (Farré et al. 2016).

Supplemental References

Blanchette M, Kent WJ, Riemer C, Elnitski L, Smit AF, Roskin KM, Baertsch R, Rosenbloom K, Clawson H, Green ED et al. 2004. Aligning multiple genomic sequences with the threaded blockset aligner. *Genome Res* **14**: 708-715.

Farré M, Narayan J, Slavov GT, Damas J, Auvil L, Li C, Jarvis ED, Burt DW, Griffin DK, Larkin DM. 2016. Novel insights into chromosome evolution in birds, archosaurs, and reptiles. *Genome Biol Evol* doi:10.1093/gbe/evw166.

Hansmann T, Nanda I, Volobouev V, Yang F, Schartl M, Haaf T, Schmid M. 2009. Cross-species chromosome painting corroborates microchromosome fusion during karyotype evolution of birds. *Cytogenet Genome Res* **126**: 281-304.

Harris RS. 2007. Improved pairwise alignment of genomic DNA. Vol Ph.D. The Pennsylvania State University.

Hubisz MJ, Pollard KS, Siepel A. 2011. PHAST and RPHAST: phylogenetic analysis with space/time models. *Brief Bioinform* **12**: 41-51.

Jarvis ED Mirarab S Aberer AJ Li B Houde P Li C Ho SYW Faircloth BC Nabholz B Howard JT et al. 2014. Whole-genome analyses resolve early branches in the tree of life of modern birds. *Science* **346**: 1320-1331.

Kent WJ, Baertsch R, Hinrichs A, Miller W, Haussler D. 2003. Evolution's cauldron: Duplication, deletion, and rearrangement in the mouse and human genomes. *Proc Natl Acad Sci U S A* **100**: 11484-11489.

Kim J, Larkin DM, Cai Q, Asan, Zhang Y, Ge R-L, Auvil L, Capitanu B, Zhang G, Lewin HA et al. 2013. Reference-assisted chromosome assembly. *Proc Natl Acad Sci U S A* **110**: 1785-1790.

Langmead B, Salzberg SL. 2012. Fast gapped-read alignment with Bowtie 2. *Nat Meth* **9**: 357-359.

Nishida C, Ishijima J, Kosaka A, Tanabe H, Habermann FA, Griffin DK, Matsuda Y. 2008. Characterization of chromosome structures of Falconinae (Falconidae, Falconiformes, Aves) by chromosome painting and delineation of chromosome rearrangements during their differentiation. *Chromosome Res* **16**: 171-181.
